# Assessment of the Interaction of Acetylcholinesterase Binding with Bioactive Compounds from Coffee and Coffee Fractions Digested *In Vitro* in the Gastrointestinal Tract

**DOI:** 10.1021/acs.jafc.4c05435

**Published:** 2024-10-04

**Authors:** Joanna Grzelczyk, Horacio Pérez-Sánchez, Miguel Carmena-Bargueño, Alejandro Rodríguez-Martínez, Grażyna Budryn

**Affiliations:** † Institute of Food Technology and Analysis, Faculty of Biotechnology and Food Sciences, Lodz University of Technology, Lodz 90-537, Poland; ‡ Structural Bioinformatics and High-Performance Computing Research Group (BIO-HPC), Computer Engineering Department, Universidad Católica de Murcia (UCAM), Guadalupe, Murcia 30107, Spain

**Keywords:** acetylcholine, docking simulation, coffee, ITC, acetylcholinesterase

## Abstract

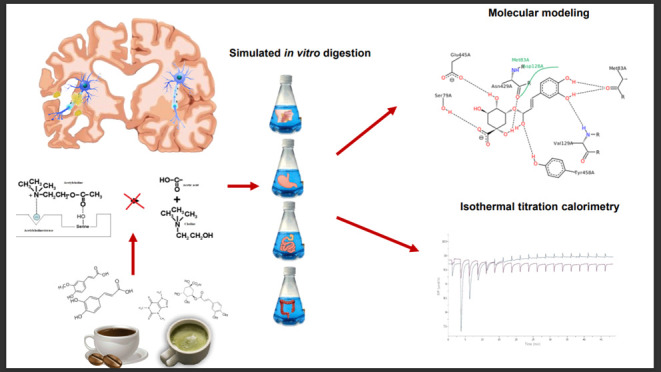

The aim of the study was to evaluate the degree of acetylcholinesterase (AChE) inhibition by green and light- and dark-roasted coffee extracts and their fractions after digestion in a simulated gastrointestinal tract. The analysis was carried out using isothermal titration calorimetry, molecular docking, and dynamics simulations. The results showed that 3-*O*-caffeoylquinic acid binds strongly to AChE through hydrogen interactions with the amino acids ARG289A, HIS440A, and PHE288A and hydrophobic interactions with TYR121A in the active site of the enzyme. The Robusta green coffee extract (Δ*G* = −35.87 kJ/mol) and dichlorogenic acid fraction (Δ*G* = −19–29 kJ/mol) showed the highest affinity. Dichlorogenic acids (3,4-*O*-dicaffeoylquinic acid, 4,5-*O*-dicaffeoylquinic acid, and 3,4-*O*-dicaffeoylquinic acid) have high affinity for AChE as single compounds (Δ*G*(ITC) = −48.99–55.36 kJ/mol, Δ*G*(LF/AD) = −43.38–45.38 kJ/mol). The concentration necessary to reduce AChE activity by 50% amounted to 0.22 μmol/μmol chlorogenic acids to the enzyme.

## Introduction

Alzheimer’s disease (AD) is one of the most common dementias in the world. It is a partially latent disease that consists of several stages. In the first stage, the disease is asymptomatic. Invisible lesions start with small changes in the brain. This disease manifests itself in increased loss of nerve cells, gray matter, and white matter compared to the brain of a healthy person. The main region of the brain where the greatest neuronal atrophy occurs is the prefrontal cortex, and the development of tau protein pathology begins in the hippocampus, initiating the development of AD and affecting the deterioration of cognitive functions. Such a disorder manifests itself as the so-called subjective deterioration of cognitive functioning, which does not significantly affect everyday life, is difficult to recognize due to its mild course, and may cause hidden symptoms lasting up to 4 years.^1,2^ In subsequent stages of AD development, the volume of the prefrontal cortex, corpus striatum, and hippocampal neurogenesis increases. These changes lead to disruption of neuronal connections, loss of synapses, and a reduction in the level of functional neurons. At this stage, neurons responsible mainly for creating new memories are damaged, contributing to difficulties in remembering new information.^3,4^ As the disease progresses, patients may notice increasing difficulties in performing daily household chores and planning and carrying out tasks, disorientation in the field, forgetting familiar places, losing objects, and speech disorders, manifested by forgetting words.^5^

In the last stages of the disease, an important role in the course of AD is most likely played by the extracellular deposition of amyloid and neurofibrillary tangles, which leads to neuronal apoptosis. These changes cause a sudden intensification of symptoms of the disease. They affect the proper functioning of the sick person in everyday life.^2,4,6^ The main enzyme responsible for AD is acetylcholinesterase.

Acetylcholinesterase (AChE) is an enzyme that catalyzes the hydrolysis of acetylcholine (ACh).^7^ The catalyzed hydrolysis of ACh prevents its accumulation in the brain by removing excess of the neurotransmitter from the synaptic cleft.^8,9^ As a result of the activity of choline acetyltransferase in cholinergic neurons, ACh is synthesized from acetyl coenzyme A (acetyl–CoA), a product of glycolysis, and from choline returned to neurons via the high-affinity choline transporter. As a result of depolarization, ACh is released from presynaptic cells and then hydrolyzed again by AChE.^10−12^ AChE is located in the central and peripheral nervous system. The active site of the enzyme consists of a catalytic triad: a base, histidine; a nucleophile, serine; and an acid, in this case, glutamic acid. The enzyme is responsible for the direct hydrolysis of choline esters using a covalent hydrogen bond from serine to histidine. The excessive AChE activity and hydrolysis of choline esters appearing, for example, in Alzheimer disease (AD) contribute to the reduction of ACh concentration below normal, lead to the accumulation of β-amyloid and aggregation of T protein, and affect the formation of fibrosis and inflammation.^13,14^ The action of AChE cannot be blocked completely in an irreversible manner. The proof is the action of organophosphate substances, contributing to the irreversible inhibition of the active site of AChE by phosphorylation. Organophosphate compounds bind covalently with the serine residue that results in the excessive accumulation of ACh in cholinergic synapses and may lead to death.^15−17^

The therapies aimed at reducing the symptoms of AD are based on the use of AChE inhibitors. AChE inhibitors delay the development of degenerative changes and protect against the expression of phosphorylated tau protein by preventing the formation of β-amyloid. Unfortunately, long-term use of synthetic AChE inhibitors causes side effects such as heart disorders, severe gastrointestinal and liver disorders, diarrhea, insomnia, fainting, nausea and vomiting, muscle cramps, feeling of fatigue, headache and dizziness, and weight loss.^18−20^ Therefore, it is important to evaluate food constituents as natural, reversible, and moderate AChE inhibitors. Epidemiological studies have shown that regular coffee consumption is associated with slower cognitive decline, although not all studies provide consistent results, which may be due to different doses of coffee consumed and the severity of neurological disorders. The aim of the study was to determine the affinity of coffee extracts for AChE using thermodynamic analysis and molecular simulations, depending on the degree of coffee roasting and the digestion phase.

## Materials and Methods

### Materials and Reagents

Acetylcholinesterase from *Electrophorus electricus* (electric eel) and acetylcholine chloride were purchased from Sigma-Aldrich (St. Louis, MO), and nylon filters were purchased from Chromacol (Herts, U.K.). All other reagents were analytical grade and were purchased from Chempur (Piekary Slaskie, Poland). The coffee beans, green Arabica (*Coffee arabica* L.), variety Brazil Cerrado, and green Robusta (*Coffea canephora* L.), variety India Cherry, were purchased from Bero Polska (Gdynia, Poland).

The preparation of coffee extracts and their fractions was described in the previous work of Grzelczyk et al.^21^ In short, the green beans of Arabica and Robusta were roasted and ground and aqueous extracts were obtained using the laboratory pressure method. Next, the extracts were filtered and lyophilized. Then, freeze-dried extracts from green and light- and dark-roasted beans were fractionated using centrifugal partition chromatography (Spot Prep II system (Armen, France), CPC). Fractionation of the extracts and their preliminary purification using the CPC method allowed us to obtain concentrations of the mentioned substances in the range of 8–50 g/100 g d.b. for monochlorogenic acids, 3–22 g/100 g d.b. for dichlorogenic acids, and 10–21 g/100 g d.b. for caffeine. The remaining part of the fractions consisted of polymeric components (proteins, carbohydrates, Millard reaction products) and to a small extent the components from neighboring fractions.^21^ They were characterized by a high content of isolated compounds. For monochlorogenic acids in Robusta, the value amounted to 50.41 g/100 g d.b. for green, 36.91 g/100 g d.b. for the light-roasted, and 18.13 g/100 g d.b. for the dark-roasted beans. Arabica monochlorogenic acid fractions contained about 1.7–2 times less of these compounds: 29.27 g/100 g d.b. for green, 7.56 g/100 g d.b. for the light-roasted, and 8.91 g/100 g d.b. for the dark-roasted. The content of dichlorogenic acids was half that of the monochlorogenic acids, and their content decreased with a higher degree of roasting. The content of dichlorogenic acids in green coffee was 22.69 g/100 g d.b. in Robusta and 13.2 g/100 g d.b. in Arabica; in light-roasted, it was 11.08 g/100 g d.b. in Robusta and 8.76 g/100 g d.b. in Arabica, and in dark-roasted, it was 5.21 g/100 g d.b. and 3.09 g/100 g d.b., respectively. The caffeine content in the isolated fractions from green coffee was 21.9 g/100 g d.b. in Robusta and 12.95 g/100 g d.b. in Arabica; in light-roasted, it was 21.00 g/100 g d.b. and 12.15 g/100 g d.b., and in dark-roasted, it was 18.14 g/100 g d.b. and 10.00 g/100 g d.b., respectively. Caffeine is a stable compound and is slightly degraded on roasting, while the already light roasting of coffee produced MRPs such as 5-HMF and AK at concentrations of 1.4–15.1 and 1.2–14.6 μg/100 g d.b., respectively.^22^

Digestion was carried out according to the method of Grzelczyk et al.^23,24^ In short, the lyophilized coffee extracts and their fractions were dissolved in water (green or light- or dark-roasted coffee).

- The “gastric phase”: In 1.7 pH (5 mol L, HCl), pepsin was added and incubated (stirring 20 rpm, 37 °C, 1.5 h.) Next, a solution of NaHCO_3_ was added (0.1 mol/L; pH 7.5).
- The “small intestine” phase: 150 mL of Gibson’s solution was added to the sample,^25^ and the pH was corrected (pH 6.5, 1 mol L^–1^ HCl). Next, pancreatin and bile salt were added and incubated for 3 h.
- The “large intestine” phase: With another 3 h of incubation, the Jarro-Dophilus EPS vaccine was added (9 strains added at a dose of 0.625 × 109 cfu). 10 mL of sterilized and cooled saline (0.9% aqueous NaCl solution) was aseptically added to the bacterial inoculum.
- The “colon” phase: samples were incubated for 9h.

After collection, each sample was heated in a water bath (65 °C, 15 min) to inactivate the enzymes and frozen at −80 °C for analysis.^23,24^

### Isothermal Titration Calorimetry (ITC)

Protein–ligand interaction analysis was performed by isothermal titration calorimetry (ITC) using a MicroCal PEAQ-ITC200 instrument (Malvern, Worcestershire, U.K.). The analysis was conducted according to the procedure of Grzelczyk and Budryn^26^ with some modifications. The measuring cell of the calorimeter with a capacity of 0.2 mL was filled with a degassed AChE solution (20 μmol/L, enzyme diluted with ultrapure water). The syringe of the device was filled with aqueous solutions of coffee extracts or fractions isolated from green and roasted coffee extracts (rich in monochlorogenic acids, dichlorogenic acids, or caffeine, with a concentration of 10 mmol/L based on the main component of the fraction). Coffee extracts and their fractions after *in vitro* digestion in a simulated system at an appropriate point of the *in vitro* gastrointestinal tract were analyzed at a concentration of 0.2 mmol/L with a concentration of the enzyme amounting to 0.1 μmol/L with or without probiotic bacteria. The solutions of coffee bioactive compounds were gradually injected into the measuring cell in 2 μL portions, where the tested compounds were diluted in the AChE solution and interacted with the enzyme. The research assumed the mass of the AChE monomer corresponding to the form found in body fluids. The analysis was carried out at a temperature of 36.6 °C (representing the temperature of the human body) with constant stirring (307 rpm). The time between injections was 150 s. A total of 19 injections were performed within 50 min.

During the experiments, enthalpy changes (Δ*H*), entropy changes (Δ*S*), and Gibbs free energy changes based on the Gibbs equation, also described as affinity (Δ*G*), the dissociation constant (*K*_d_), the complexation (association) constant (*K*_a_), and the inhibition constant of the enzymatic reaction (*K*_i_), were determined. The *K*_i_ values of ACh hydrolysis catalyzed by AChE in the presence of inhibitors were calculated using the Michaelis–Menten equation and a model of the enzymatic reaction in the presence of an inhibitor. Data were calculated using MicroCal PEAQ-ITC Analysis software.^26^

### Molecular Docking

The characteristic X-ray crystal structure of the AChE enzyme with PDB code 1EVE from the Protein Data Bank (PDB) database (http://www.rcsb.org/pdb) was selected for analysis. Then, a full-atom model of the enzyme was prepared by the Protein Preparation Wizard available in the Maestro software suite^27^ by adding hydrogen atoms and considering protonation states. The chemical structures of chlorogenic acids and caffeine molecules were built up manually using the Gasteiger scheme.^28^ Docking of bioactive coffee compounds into the prepared enzyme structure model was performed using Lead Finder docking software^29^ and AutoDock Vina^30^ with its respective default binding mode parameters. The size of the ligand docking grid was set to extend 30 Å in each direction from the geometric center of the molecule for each individual docking simulation. The scoring function used in the calculations took into account the Lennard-Jones potential, hydrogen bonds, electrostatic interactions, hydrophobic stabilization, rotational bonds, and internal energy of the ligand. Software PoseView^31^ was used next to allow visualization of the predicted protein–ligand interactions.

A sequential docking was performed for each binding mode of the compounds using both docking algorithms. For this, the compound pose obtained by the first docking (Step 1) is included in the structure of the protein for the second docking (Step 2). This procedure was repeated for the third docking to the final binding pose of the three compounds (Step 3). Consequently, this process yielded 12 binding poses through three distinct steps.

### Molecular Dynamics Simulations

We conducted molecular dynamics (MD) simulations on the complexes with the binding poses of higher affinity results of the three compounds to validate their role as acetylcholinesterase (AChE) inhibitors in a water-based system over time.

MD simulations lasting 100 ns (ns) were initiated for each complex. Initially, the ligand’s topology was generated using an automated script leveraging ACPYPE,^32^ which applied an AMBER99SB force field to the ligand. Subsequent steps of the MD simulations were executed using GROMACS 2022.3,^33^ facilitated by the Picasso server (https://www.scbi.uma.es/) with GPU (NVIDIA A100-SXM4-40GB) and 4 GB of RAM. The process commenced with protein topology creation using the gmx pdb 2gmx command, utilizing also AMBER99SB as the force field. Subsequently, a simulation dodecahedron box was defined (centering the protein with 0.9 nm from the box edge) and solvated using the TIP3P water model, and Na^+^ and Cl^–^ ions were added. A 2000 ps energy minimization phase was conducted, followed by a single NvT equilibration stage of 50.000 ps and sequential five NPT equilibration stages of 50.000 ps each. The dynamics were then simulated for 100 ns, with a temperature of 300 K and using the Verlet and Nose–Hoover algorithms, for the thermostat and barostat, respectively. Finally, the resultant trajectory was extracted at various frames to assess the ligands’ stability and movement relative to the binding pose. Also, periodic boundary conditions (PBCs) were applied using the trjconv command with the -pbc parameter indicated. Finally, MM-PBSA analysis was performed by the g_mmpbsa tool.^34,35^ In all the analyses performed, the ligand is considered to be all of the heavy atoms of the three ligands of the combination; meanwhile, the protein is considered to be all of the atoms belonging to the structure.

### Statistical Analysis

Statistical analysis was based on the determination of the average values of nine measurements and their standard deviation, as well as one-way analysis of variation (ANOVA), using Statistic 10.0 software at the significance level *P* < 0.05. Significance was defined at *p* ≤ 0.05.

## Results and Discussion

### Interactions and Affinity of Coffee Extracts for AChE

In the previous article by Grzelczyk and Budryn,^26^ the degree of inhibition of AChE activity by individual chlorogenic acids and coffee extracts was assessed. The interactions and their thermodynamic parameters were determined calorimetrically. The ITC analysis showed the most stable complex and strong interactions of 3-*O*-caffeoylquinic acid (3-CQA) with AChE, Δ*H* = −28.13 kJ/mol and Δ*G* = −30.50 kJ/mol. The Robusta green coffee extract showed the highest affinity and enthalpy change of interaction with AChE (Δ*H* = −8.17 kJ/mol, Δ*G* = −35.87 kJ/mol) and was also the most effective AChE inhibitor.^25^ In the present study, we investigated how digestion simulated in the digestive system *in vitro* changes the affinity of coffee infusions for AChE. To better understand the impact of quantitative changes in individual coffee components on changes in the affinity for the enzyme, we performed the simulation of docking of chlorogenic acids, caffeine, and dihydrocaffeic acid (one of the main metabolites of chlorogenic acids) to AChE. The simulation confirmed that the docked substances characteristic for coffee brews bound in the active site of the enzyme.^34^ The observed higher affinity of diesters (dichlorogenic acids) compared to monoesters, expressed as a higher negative value (Table 1), may result from the lower flexibility of the smaller molecules of monoesters when binding to AChE as well as the lower number of moieties capable of binding with the amino acids of the enzyme active side. AChE is characterized by a narrow pocket that requires structure adjustments in order for the ligand to reach the active site.^36^

**Table 1:** Binding Energy of Interactions of AChE with Hydroxycinnamic Acids, Chlorogenic Acids, and Caffeine Calculated from LeadFinder and AutoDock Vina Docking Simulation Software

compound	Δ*G* (kJ/mol) (LF/AD)
caffeic acid	–26.04/–28.45
ferulic acid	–26.62/–30.54
3-*O*-caffeoylquinic acid	–47.31/–37.05
4-*O*-caffeoylquinic acid	–46.31/–37.56
5-*O*-caffeoylquinic acid	–45.22/–39.48
3,4-*O*-dicaffeoylquinic acid	–55.36/–45.13
4,5-*O*-dicaffeoylquinic acid	–48.99/–45.38
3,5-*O*-dicaffeoylquinic acid	–54.39/–43.38
dihydrocaffeic acid	–25.74/–43.93
caffeine	–20.76/–26.78

**Figure 1:**
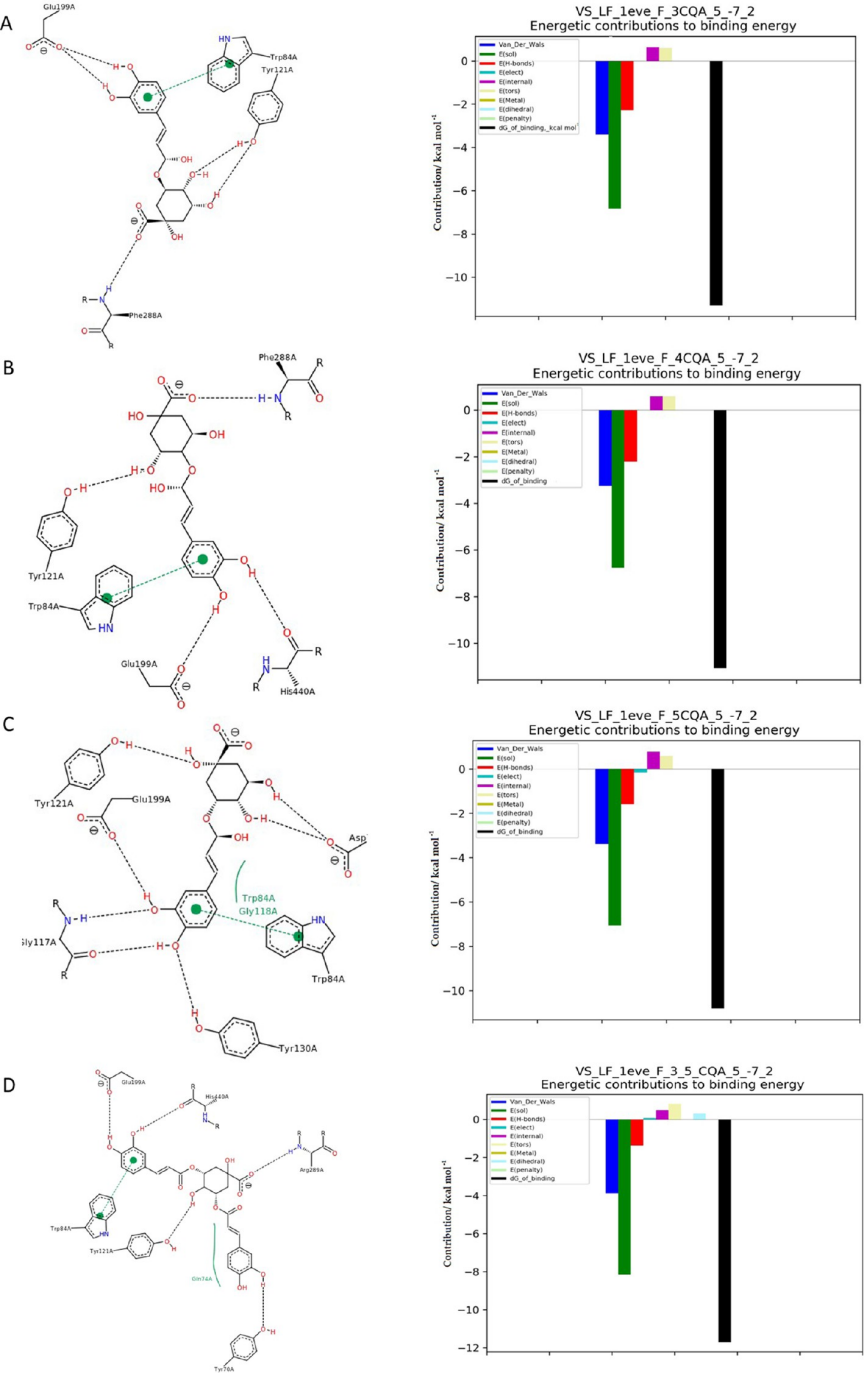

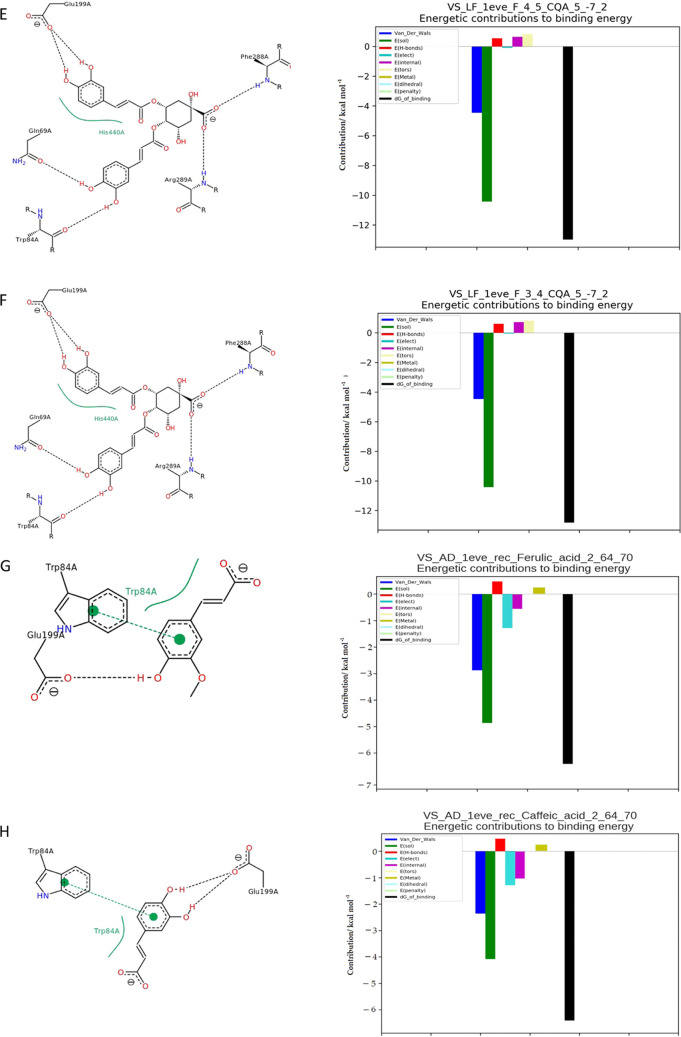

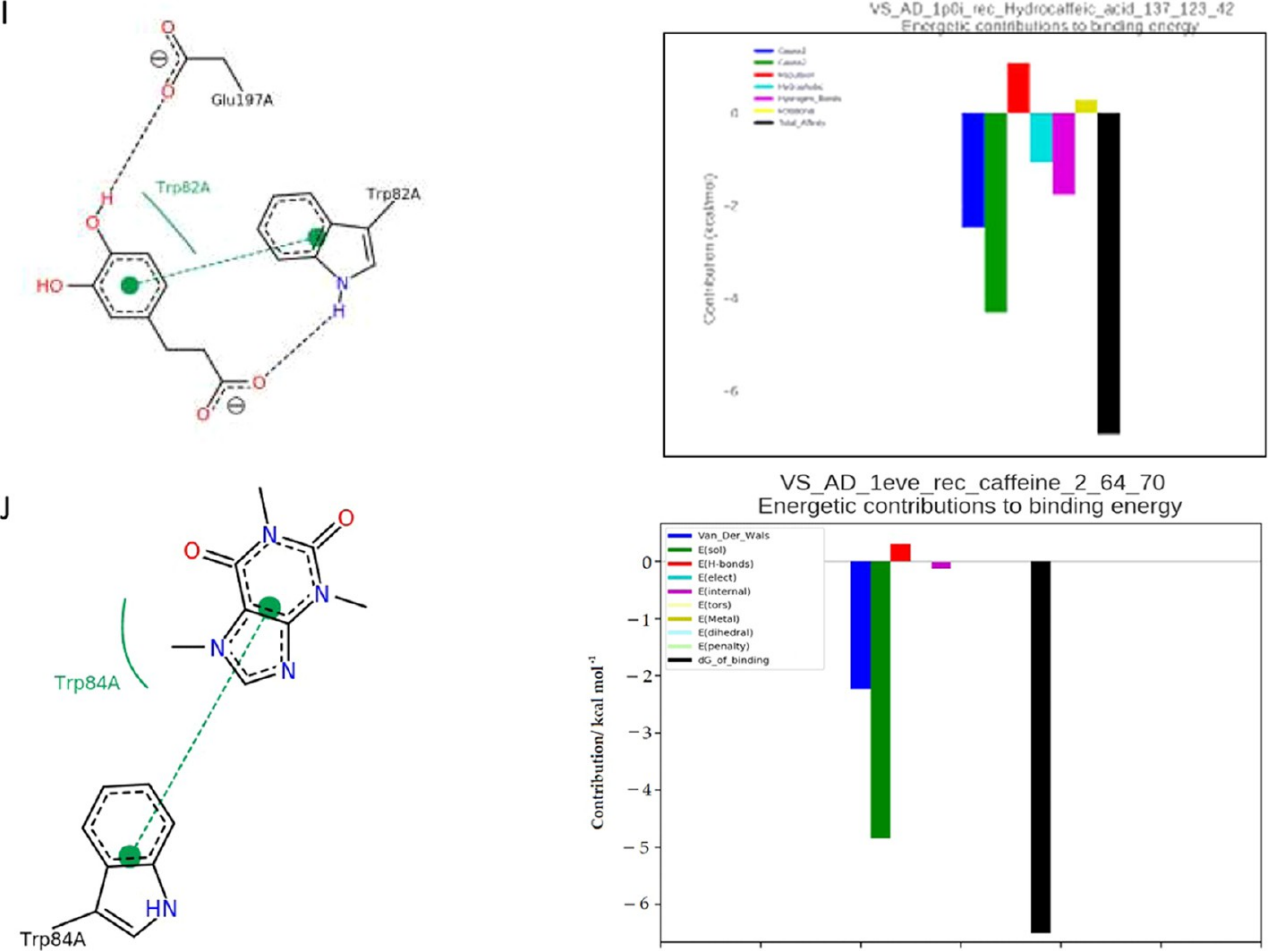
Docking simulation results by software with the best score for each compound. On the left, two-dimensional (2D) models, interactions of AChE with chlorogenic acids, caffeine, and dihydrocaffeic acid; black dashed line: hydrogen bonds, and green dashed line: hydrophobic interactions. On the right side, the energy values of the interactions that make up the total interaction energy of AChE and (A) 3-CQA, (B) 4-CQA, (C) 5-CQA, (D) 3,5-DCQA, (E) 4,5-DCQA, (F) 3,4-DCQA, (G) ferulic acid, (H) caffeic acid, (I) dihydrocaffeic acid, and (J) caffeine. Contribution of van der Waals forces: dark blue; free energy of solvation: green, hydrogen bonds: red, electrostatic energy: blue, energy of surface contacts: purple, energy related to entropy loss due to rotation: gray yellow, cationic interactions: gray green, local steric effects: light blue, energy penalty for cavity site opening: turquoise, and predicted total binding energy: black.

Figure 1A shows that 3-CQA binds strongly to AChE through hydrogen bonds with the amino acids ARG289, HIS440, and PHE288 and hydrophobic interactions with TYR121. HIS440 is a part of the catalytic triad in the active side of AChE. As a result of the interaction of 4-*O*-caffeoylquinic acid (4-CQA) with the enzyme, there were also hydrogen interactions with the amino acids ARG289 and PHE288 and hydrophobic interactions with ASP72 (Figure 1B).

The 5-isomer exhibited analogous interactions, except that additional hydrophobic interactions were formed with SER122 located in the catalytic triad and TYR121; therefore, 5-O-caffeoylquinic acid (5-CQA) was characterized by a slightly higher affinity (Table 1). Caffeic acid formed two hydrogen bonds with GLU199 of the active site characterized by positive energy, lowering the total interaction energy, which explains the low affinity for AChE (Figure 1H). There were also π–π and hydrophobic interactions with TRP84. A similar tendency was observed for caffeine, which had the lowest affinity for AChE, and the interaction energy in the case of the alkaloid resulted mostly from π–π and hydrophobic interactions with TRP84 (Figure 1J). The most effective AChE inhibitors were caffeic acid diesters, as confirmed by docking simulation. Diesters were characterized by high van der Waals forces, exceeding −4 kcal mol, and high solvation free energy (Figure 1D–F). Depending on the location of hydroxyl groups in the aromatic ring, forming ester links in diesters, the following hydrophobic interactions were observed: 3,4-di-*O*-caffeoylquinic acid (3,4-CQA) and 4,5-di-*O*-caffeoylquinic acid (4,5-CQA), hydrophobic interactions with HIS440A, and 3,5-di-*O*-caffeoylquinic acid (3,5-CQA), hydrophobic interactions with GLY74.

The studies showed statistically significant differences between the interactions of coffee extracts with AChE before and after digestion. *In vitro* digestion of coffee extracts in the stomach resulted in an intense increase in the analyzed parameters compared to extracts before digestion. For example, in the extracts from green beans, the binding constant (*K*_a_) after digestion in the gastric phase increased from 6.71 × 10^3^ and 11.50 × 10^3^ L/mol^26^ to the range of 116.55–113.51 L/mol (Table 2), for green Arabica and Robusta, respectively. With the degree of coffee roasting, some of the hydroxycinnamic and (HCAs) chlorogenic acids (CHAs) bind to melanoidins as well as other compounds such as proteins and carbohydrates, which has a significant impact on the bioavailability of these phenolic compounds. During the digestion of coffee extracts in the stomach, polyphenolic compounds are released from the links with melanoidins under the influence of acid and alkaline hydrolysis.^37,38^ For example, Robusta coffee extracts showed the content of HCAs, including chlorogenic acids (CHAs) in the range of 5–31 g/100 g d.b., with Arabica showing 3–17 g/100 g d.b. and caffeine in the range of around 6 g/100 g d.b. for Robusta and 3–4 g/100 g d.b. for Arabica. The contents of acrylamide and 5-hydroxymethylfurfural increased during roasting. After digestion in the stomach, there was a decrease in polyphenols, and for Robusta coffee extracts, the content of HCAs, including CHAs, was in the range of 1–2 g/100 g d.b., and for Arabica, it was 0.75–2 g/100 g d.b.; caffeine was around 4 g/100 g d.b. (Robusta) and 2–3 g/100 g d.b. (Arabica).^23^ Polyphenols contained in coffee are partially absorbed in the stomach without prior hydrolysis, through passive transport; this applies to both mono- and diesters of caffeic acid with quinic acid.^38^ The observed bioavailability of polyphenols at this stage of digestion was very low, which could be due to the formation of additional CHAs and melanoidin compounds in a highly acidic environment.^23^ Farah et al.^40^ showed that during *in vivo* coffee digestion, especially at the stomach stage, the content of free CHAs decreased, which results in reduced bioavailability of these compounds.^39^ Research by Costa et al.^41^ showed that the degree of CHA adsorption by dietary fiber changes with pH, reaching a maximum at pH 2.0, and this phenomenon is largely reversible at pH 7.0.^41^ The pH lowers in the stomach, which causes various interactions (ester bonds, hydrogen bonds) between melanoidins included in the coffee fiber and CHAs.^42^ In the stomach, these compounds are less available for absorption but remain active as enzyme-complexing compounds, and the increase in the complexation constant may be due to partial hydrolysis of the polymeric material at low pH in the stomach, which promotes binding to AChE. The affinity of coffee extracts digested in the stomach for the enzyme is related to both the location and the number of hydroxyl groups and the hydrophobicity of CHAs, which are higher for diCHAs. It is worth mentioning that in the first 2 h after coffee extract administration, the ratio of diesters to monoesters of caffeic acid in the plasma may be higher than in the extract itself as a result of the different dynamics of absorption of the individual CHAs, which relatively increases the affinity of chlorogenic acids for AChE.^43,44^ The concentration of caffeine which is absorbed in the stomach by about 20% was higher in Robusta extracts, and it may be also the reason for better AChE inhibition parameters by Robusta extracts.^44^

**Table 2:** ITC Parameters of Interactions of Coffee Extracts Digested *In Vitro* with AChE and Their Activity as Enzyme Inhibitors^a^

type of coffee extract	*K*_d_ (nmol/L)	*K*_a_ *10^3^ (L/mol)	Δ*H* (kJ/mol)	Δ*G* (kJ/mol)	Δ*S* [J/(mol K)]	inhibitor activity (%)	*K*_i_ (μmol/L) *K*_M_ Ach 48.5 μmol/L	IC_50_ (μmol) inhibitor concentrations: μmol AChE
Gastric Phase								
green arabica	8.58 ± 1.02^a^	116.55 ± 3.39^a^	–170.40 ± 3.15^a^	–30.06 ± 2.10^a^	–453.96 ± 8.39^a^	88.99 ± 3.28^a^	0.24 ± 0.02^a^	6.61 ± 0.18^a^
light-roasted arabica	8.37 ± 1.11^a^	119.47 ± 3.25^a^	–172.54 ± 3.45^a^	–30.14 ± 2.25^a^	–460.60 ± 9.25^a^	87.93 ± 3.10^a^	0.63 ± 0.03^b^	6.69 ± 0.10^b^
dark-roasted arabica	6.64 ± 1.20^b^	150.60 ± 3.05^b^	–190.92 ± 3.09^b^	–30.73 ± 2.15^a^	–518.15 ± 9.21^b^	80.86 ± 3.39^a^	1.11 ± 0.03^c^	7.27 ± 0.35^c^
green robusta	8.81 ± 1.19^a^	113.51 ± 2.10^a^	–147.50 ± 3.82^c^	–30.02 ± 2.51^a^	–380.01 ± 11.12^c^	97.68 ± 2.39^a^	0.12 ± 0.02^a^	4.65 ± 0.55^d^
light-roasted robusta	8.41 ± 1.10^a^	118.91 ± 2.45^a^	–166.89 ± 3.55^a^	–29.94 ± 2.33^b^	–442.99 ± 9.44^d^	92.05 ± 5.35^a^	0.62 ± 0.01^b^	4.94 ± 0.35^e^
dark-roasted robusta	8.76 ± 1.09^a^	114.16 ± 2.35^a^	–168.90 ± 3.39^a^	–30.02 ± 2.18^a^	–449.22 ± 9.45^d^	84.43 ± 1.45^b^	1.00 ± 0.05^c^	5.38 ± 0.45^f^
Small Intestine Phase								
green arabica	12.12 ± 1.15^c^	82.51 ± 2.91^c^	96.72 ± 2.34^d^	–22.40 ± 1.10^c^	385.30 ± 7.09^c^	8.30 ± 0.15^c^	0.79 ± 0.05^d^	70.89 ± 1.02^a^
light-roasted arabica	11.40 ± 1.10^c^	87.72 ± 2.45^c^	136.72 ± 3.12^c^	–23.40 ± 1.05^c^	517.20 ± 6.24^b^	6.62 ± 0.29^d^	0.69 ± 0.02^b^	88.85 ± 1.06^g^
dark-roasted arabica	41.72 ± 1.25^e^	23.96 ± 1.39^d^	112.69 ± 3.44^c^	–28.85 ± 1.15^b^	457.83 ± 9.53^d^	11.66 ± 1.10^d^	1.82 ± 0.01^e^	50.46 ± 1.13^h^
green robusta	16.80 ± 1.09^d^	59.52 ± 2.05^d^	103.41 ± 3.67^d^	–23.24 ± 1.20^c^	409.67 ± 8.01^d^	27.03 ± 1.05^a^	0.76 ± 0.01^d^	16.82 ± 0.39^d^
light-roasted robusta	10.32 ± 1.10^c^	96.90 ± 2.15^e^	136.91 ± 3.32^c^	–23.40 ± 1.29^c^	518.56 ± 9.25^b^	14.74 ± 1.15^b^	0.56 ± 0.02^b^	30.83 ± 01.44^e^
dark-roasted robusta	13.82 ± 1.22^c^	72.36 ± 2.33^e^	101.74 ± 3.11^d^	–20.05 ± 1.25^c^	393.96 ± 9.59^c^	13.46 ± 1.25^d^	1.11 ± 0.01^c^	33.77 ± 1.94^d^
Large Intestine Phase after 4 h								
green arabica	16.60 ± 1.28^d^	60.24 ± 1.55^e^	101.74 ± 6.25^d^	–34.29 ± 1.10^e^	440.01 ± 6.12^d^	32.75 ± 2.22^c^	0.25 ± 0.01^a^	17.96 ± 0.45^i^
light-roasted arabica	76.18 ± 1.25^f^	13.13 ± 1.15^f^	139.00 ± 2.35^c^	–24.45 ± 1.28^c^	528.72 ± 6.25^b^	32.39 ± 0.45^d^	0.89 ± 0.05^d^	18.16 ± 0.15^g^
dark-roasted arabica	97.40 ± 1.05^g^	10.27 ± 1.20^f^	115.49 ± 2.18^d^	–23.82 ± 1.25^c^	450.63 ± 6.94^d^	35.18 ± 0.41^e^	1.05 ± 0.04^c^	16.72 ± 0.13^h^
green robusta	21.25 ± 1.69^e^	47.06 ± 1.38^d^	124.35 ± 2.33^c^	–21.81 ± 1.39^c^	472.78 ± 6.18^d^	37.21 ± 0.35^e^	0.48 ± 0.03^a^	12.22 ± 0.11^d^
light-roasted robusta	35.72 ± 1.19^e^	28.00 ± 1.35^d^	126.02 ± 1.45^c^	–20.47 ± 1.45^c^	473.87 ± 9.45^d^	41.11 ± 0.29^d^	0.88 ± 0.02^d^	11.06 ± 0.93^e^
dark-roasted robusta	66.19 ± 1.35^f^	15.11 ± 1.51^f^	255.39 ± 2.15^e^	–18.88 ± 1.09^f^	887.20 ± 9.09^e^	26.58 ± 0.55^d^	1.12 ± 0.02^c^	17.10 ± 0.44^d^
Large Intestine Phase after 10 h								
green arabica	12.36 ± 1.39^c^	80.91 ± 1.45^e^	100.90 ± 6.45^d^	–21.65 ± 1.11^c^	396.40 ± 4.39^c^	10.43 ± 0.45^c^	0.97 ± 0.01^f^	56.41 ± 1.09^i^
light-roasted arabica	18.25 ± 1.33^d^	54.79 ± 1.39^d^	106.34 ± 5.81^d^	–22.19 ± 1.15^c^	415.77 ± 4.45^d^	4.60 ± 0.15^d^	1.52 ± 0.02^c^	127.85 ± 1.45^g^
dark-roasted arabica	36.18 ± 1.34^e^	27.64 ± 1.44^d^	135.65 ± 5.45^c^	–21.65 ± 1.10^c^	508.81 ± 4.55^b^	11.46 ± 0.10^c^	3.09 ± 0.02^g^	51.33 ± 1.15^h^
green robusta	22.75 ± 1.39^e^	43.96 ± 1.38^d^	135.65 ± 6.34^c^	–21.65 ± 1.09^c^	508.81 ± 4.45^b^	21.78 ± 0.10^f^	0.94 ± 0.05^f^	20.87 ± 0.19^d^
light-roasted robusta	20.45 ± 1.35^e^	48.90 ± 1.29^d^	147.38 ± 6.25^c^	–21.90 ± 1.10^c^	547.54 ± 3.39^b^	19.19 ± 0.39^f^	1.09 ± 0.04^c^	23.69 ± 0.95^e^
dark-roasted robusta	57.46 ± 1.25^f^	17.40 ± 1.45^f^	95.46 ± 6.11^d^	–21.65 ± 1.15^c^	390.17 ± 4.55^c^	31.00 ± 1.45^e^	2.14 ± 0.02^h^	14.66 ± 0.45^d^
Large Intestine Phase + probiotic bacteria after 4 h								
green arabica	1.52 ± 0.45^h^	657.89 ± 9.85^g^	118.91 ± 2.09^d^	–22.65 ± 1.45^c^	457.89 ± 9.19^d^	83.39 ± 1.25^g^	0.42 ± 0.01^a^	7.05 ± 0.39^i^
light-roasted arabica	1.49 ± 0.18^h^	671.14 ± 9.55^g^	116.39 ± 2.15^d^	–22.73 ± 1.35^c^	450.03 ± 9.54^d^	50.84 ± 1.15^g^	0.58 ± 0.01^b^	14.31 ± 1.12^j^
dark-roasted arabica	1.45 ± 0.20^h^	689.66 ± 9.45^g^	127.28 ± 2.05^c^	–23.45 ± 1.25^c^	487.55 ± 9.45^d^	62.12 ± 1.15^h^	0.99 ± 0.02^f^	9.47 ± 0.19^h^
green robusta	1.32 ± 0.15^h^	757.58 ± 9.35^h^	127.28 ± 2.09^c^	–23.03 ± 1.25^c^	486.19 ± 9.52^d^	83.39 ± 1.10^f^	0.35 ± 0.02^a^	5.45 ± 0.18^d^
light-roasted robusta	1.27 ± 0.10^h^	787.40 ± 9.30^h^	105.09 ± 2.15^c^	–23.11 ± 1.33^c^	414.68 ± 9.10^d^	50.84 ± 1.25^g^	0.44 ± 0.01^a^	8.94 ± 0.55^e^
dark-roasted robusta	1.12 ± 0.09^h^	892.86 ± 9.54^i^	142.77 ± 2.10^d^	–23.45 ± 1.40^c^	537.65 ± 9.15^b^	62.12 ± 1.45^g^	0.85 ± 0.01^d^	7.32 ± 0.59^d^
Large Intestine Phase + probiotic bacteria after 10 h								
green arabica	1.21 ± 1.15^h^	826.45 ± 2.96^i^	96.72 ± 2.25^d^	–24.07 ± 1.15^c^	390.71 ± 2.95^c^	31.34 ± 1.10^g^	0.52 ± 0.02^b^	18.77 ± 1.57^i^
light-roasted arabica	1.23 ± 0.39^h^	813.01 ± 2.45^i^	106.43 ± 2.45^d^	–30.61 ± 1.25^e^	443.26 ± 3.29^d^	38.75 ± 1.09^e^	0.81 ± 0.03^d^	15.18 ± 1.89^j^
dark-roasted arabica	1.03 ± 0.95^h^	970.87 ± 9.65^j^	263.77 ± 3.55^f^	–21.86 ± 1.20^c^	923.90 ± 3.87^f^	38.82 ± 1.15^d^	0.95 ± 0.02	19.72 ± 1.45^h^
green robusta	8.84 ± 1.10^a^	113.12 ± 2.59^e^	63.22 ± 3.40^g^	–23.24 ± 1.15^c^	279.66 ± 2.76^g^	47.14 ± 1.20^c^	0.11 ± 0.01^a^	13.95 ± 0.09^d^
light-roasted robusta	6.98 ± 0.15^b^	143.27 ± 2.18^l^	72.43 ± 2.39^g^	–22.82 ± 1.20^c^	308.10 ± 2.28^c^	31.59 ± 2.25^e^	0.56 ± 0.00^b^	14.39 ± 0.28^e^
dark-roasted robusta	2.06 ± 0.45^h^	485.44 ± 2.35^k^	152.40 ± 2.95^a^	–23.66 ± 1.25^c^	569.48 ± 3.43^b^	47.14 ± 1.10^e^	0.76 ± 0.01^d^	9.64 ± 0.33^d^

a Values are expressed as mean ± standard deviation (SD); *n* = 9; different letters in one column correspond to significant differences (*P* < 0.05).

The studied coffee extracts after digestion in the stomach showed an exothermic effect of the interaction with the enzyme. The reaction enthalpy of extracts after digestion in the stomach was characterized by highly negative values, especially for dark-roasted arabica, and amounted to −190.92 kJ/mol. It can be observed that with the increase in the roasting degree, the reaction enthalpy also increased. However, the affinity (Δ*G*) of all coffee extracts for the enzyme was approximately 30 kJ/mol, and the differences were not statistically significant.

*In vitro* digestion in the stomach was followed by digestion in the small intestine, after which the binding constant of the extracts to AChE decreased, and the energetic effect changed to endothermic. The reaction enthalpy increased, ranging from 96.72 to 136.91 kJ/mol for green Arabica and light-roasted Robusta. This is related to the increase in the concentration of free CHAs after *in vitro* digestion in the small intestine.^23^ This is a result of the hydrolysis of internal esters, i.e., lactones, as well as external esters with carbohydrates, which is favored by a higher pH. The CHAs are ester-bonded most often by arabinose and also linked by a glycosidic bond.^45,46^ These types of bonds may also undergo hydrolysis in the small intestine due to the increase in pH. During digestion in the small intestine, the affinity for the enzyme slightly increased (to −20.05–28.25 kJ/mol for dark-roasted Robusta and Arabica), compared to the extract digested in the gastric phase. This is due to the presence of hydrolytic enzymes that cause the release of some polyphenols from various links with the digested matrix.^40^

After the digested coffee passed into the large intestine, the evaluated enthalpy of the interactions with AChE showed a shift to a higher endothermic effect with a lower binding constant. This suggests that hydrolysis at this stage, lasting 3 h, had a significant impact on the further release of polyphenols.^40^ At the same time, during incubation, the degradation of all CHAs was observed, while the concentration of caffeic acid doubled and the content of ferulic acid increased due to the hydrolysis of CHAs.^23^ After 3 h of digestion in the large intestine, the affinity for the enzyme of extracts from green and light-roasted coffee increased, while that of dark-roasted coffee decreased. The observed shift of the affinity between the large intestine and the colon may be caused by a slight decrease in pH to 6.5. It should be emphasized that at every stage of the digestive tract *in vivo*, CHAs are absorbed, which means that highly efficient bioabsorption of polyphenols can occur in the large intestine within a few hours of digestion, which was omitted in the *in vitro* simulation.^23,40^

The next considered stage was digestion in the colon, where the endothermic effect persisted, while the binding constant increased, with the exception of green Robusta, and the affinity of all coffee extracts for the enzyme was similar and amounted to approximately 21 kJ/mol. The presence of probiotic bacterial strains during *in vitro* digestion in the large intestine contributed to an efficient increase in the binding constant; it was even doubled compared to digestion without bacteria. Parkar et al.^47^ showed that intestinal microbiota, such as *Lactobacillus* and *Bifidobacterium*, can participate in the metabolism of CHAs to quinic acid and caffeic acid. Caffeic acid is further metabolized to 3-hydroxycinnamic acid, 3,4-dihydroxyphenylacetic acid, and 3-hydroxyphenylacetic acid and then absorbed in the intestine using monocarboxylic transporters. Higher concentrations of phenolic compounds increase the effect of AChE inhibition.^47^ The presence of probiotic bacteria in the colonic phase also promoted the affinity for the enzyme and the formation of more stable complexes of digested extracts with AChE.

Coffee extracts at all stages of digestion were characterized by a similar and low *K*_i_ value in the range of 0.11–2.14 μmol/L, for the green Robusta extract after digestion in the colon with the addition of bacteria and dark-roasted Robusta after digestion in the colon without microorganisms. As digestion progressed, the *K*_i_ value increased, while the addition of probiotic bacteria decreased the *K*_i_. The inhibition constant reflects the efficacy of the substrate (ACh) hydrolysis by the enzyme in the presence of the given inhibitor, in contrast to the IC50 value, which describes the ability of an inhibitor to inactivate the enzyme. Therefore, *K*_i_ values lower than *K*_M_ showed that the tested compounds bound to AChE as competitive inhibitors.^48^ It was also observed that during digestion in the stomach, a high AChE inhibition was recorded at 92–97% for green and light-roasted Robusta; only dark-roasted Robusta showed 84% inhibition activity. For Arabica, it ranged from 80 to 88%. As indicated by the IC50 values, the highest dose needed to reduce the enzyme activity by half occurred in the case of green Robusta, as well as light-roasted Robusta after digestion in the small intestine. The increase in CHA concentration occurring during digestion did not cause the expected decrease of IC50 values, which may result from the fact that large part of the compounds are associated with melanoidins, which makes it difficult to bind in the active site of the enzyme.^39^ The obtained results indicate that coffee extracts showed the greatest ability to inhibit AChE activity after digestion in the stomach. Among the coffee extracts at this stage of digestion, green Robusta had the lowest dose needed to reduce the enzyme activity by 50% (Table 2). This finding is of high importance because CHAs are in a relatively large part absorbed in the stomach in the *in vivo* conditions due to the action of the bilitranslocase and distributed in the intact form into the bloodstream. However, the metabolites formed from CHAs appear in the small intestine. After reaching the cecum, CHAs are hydrolyzed to caffeic acid of less inhibiting activity and then degraded to lower molecular weight compounds (hydroxybenzoic acid and m-coumaric acid).^49^

In the study by Işık and Beydemir,^49^ it was found that chlorogenic acid has a high ability to inhibit AChE activity with an IC50 value of 0.41 mmol for mmol enzyme, compared to caffeic acid, for which the IC50 amounted to 16.80 mmol. This can indicate that chlorogenic acid binds to AChE competitively to ACh, unlike caffeic acid.^36^ On the other hand, research by Pohanka and Dobes^50^ suggests that caffeine is a noncompetitive AChE inhibitor with the *K*_i_ value of 0.18 mmol/L. The authors also claim that caffeine is a 1000 times weaker AChE inhibitor compared to tacrine, which makes it much less toxic and can be safely administered in different therapeutic procedures.^51^ The results of the cited studies as well as the presented work confirm that phenolic compounds and caffeine present in coffee extracts are able to inhibit AChE activity at concentrations physiologically occurring after coffee consumption.

### Interactions of Fractions Isolated from Coffee Extracts with AChE

To more precisely indicate the groups of compounds with the greatest affinity for AChE and the inhibition of enzyme activity, three fractions were isolated from the extracts. The binding constant to AChE showed large differences depending on the coffee fraction in the range of 1.92–99.11 × 10^3^ L/mol for the monochlorogenic acid fraction from light-roasted Arabica and the caffeine fraction isolated from green Robusta (Table 3).

**Table 3:** Parameters of Interactions with AChE of Fractions Isolated from Coffee Extracts and Their Activity as AChE Inhibitors^a^

fractions of coffee extracts	*K*_d_ (nmol/L)	*K*_a_ *10^3^ (L/mol)	Δ*H* (kJ/mol)	Δ*G* (kJ/mol)	Δ*S* [J/(mol K)]	inhibitor activity (%)	*K*_i_ (μmol/L) *K*_M_ Ach 48.5 μmol/L	IC_50_ (μmol) inhibitor concentrations: μmol AChE
Green Arabica								
monochlorogenic acids	71.11 ± 2.03^a^	14.06 ± 1.45^a^	–1.50 ± 0.03^a^	–18.09 ± 0.01^a^	53.66 ± 1.45^a^	1.29 ± 0.01^a^	1.60 ± 0.01^a^	32.69 ± 0.09^a^
dichlorogenic acids	80.09 ± 2.25^b^	12.49 ± 1.05^a^	–3.63 ± 0.04^b^	–19.93 ± 0.03^a^	52.73 ± 0.02^a^	81.41 ± 0.11^b^	0.66 ± 0.02^b^	0.88 ± 0.01^b^
caffeine	26.40 ± 1.39^c^	37.88 ± 3.39^b^	–0.75 ± 0.01^c^	–9.55 ± 0.01^b^	28.47 ± 0.45^b^	10.46 ± 0.01^c^	1.62 ± 0.01^a^	3.41 ± 0.03^c^
Light-Roasted Arabica								
monochlorogenic acids	51.91 ± 1.39^d^	1.92 ± 0.45^c^	–0.46 ± 0.03^c^	–29.35 ± 0.01^c^	93.45 ± 2.39^c^	10.82 ± 0.01^c^	0.33 ± 0.02^c^	85.11 ± 0.15^d^
dichlorogenic acids	21.20 ± 1.05^c^	47.15 ± 1.24^d^	–12.27 ± 0.12^d^	–15.16 ± 0.01^a^	9.35 ± 1.18^d^	62.46 ± 0.04^d^	0.36 ± 0.01^c^	1.08 ± 0.01^b^
caffeine	24.10 ± 1.33^c^	41.48 ± 1.23^d^	–1.33 ± 0.02^a^	–19.59 ± 0.02^a^	59.07 ± 1.42^a^	54.90 ± 0.12^e^	0.64 ± 0.03^b^	10.51 ± 0.05^e^
Dark-Roasted Arabica								
monochlorogenic acids	36.99 ± 1.15^e^	27.10 ± 1.18^e^	–8.71 ± 0.03^e^	–32.53 ± 0.04^d^	77.05 ± 3.39^e^	1.12 ± 0.08^a^	0.11 ± 0.01^d^	98.61 ± 0.09^d^
dichlorogenic acids	17.18 ± 1.39^f^	58.21 ± 2.25^f^	–0.78 ± 0.02^c^	–18.51 ± 0.01^a^	57.35 ± 1.25^a^	69.00 ± 0.14^d^	0.25 ± 0.01^e^	1.49 ± 0.01^b^
caffeine	41.81 ± 1.55^g^	23.92 ± 2.15^e^	–11.72 ± 0.08^d^	–28.81 ± 0.02^c^	55.28 ± 0.04^a^	40.25 ± 0.12^f^	0.67 ± 0.03^b^	2.73 ± 0.02^c^
Green Robusta								
monochlorogenic acids	51.90 ± 0.12^d^	19.25 ± 0.45^b^	–6.32 ± 0.08^e^	–27.47 ± 0.11^c^	68.41 ± 1.29^e^	18.45 ± 0.02^c^	0.70 ± 0.02^b^	4.16 ± 0.02^c^
dichlorogenic acids	18.88 ± 3.18^f^	52.97 ± 1.18^f^	–17.58 ± 0.12^b^	–29.01 ± 0.03^d^	36.97 ± 1.45^f^	91.05 ± 0.08^g^	0.31 ± 0.01^c^	0.93 ± 0.00^b^
caffeine	10.08 ± 1.15^f^	99.21 ± 2.45^g^	–10.38 ± 0.01^d^	–28.81 ± 0.04^c^	59.62 ± 1.10^a^	2.12 ± 0.02^a^	1.67 ± 0.06^a^	40.18 ± 0.09^a^
Light-Roasted Robusta								
monochlorogenic acids	98.01 ± 1.23^h^	10.20 ± 1.49^a^	–1.06 ± 0.04^a^	–14.91 ± 0.01^a^	44.80 ± 1.10^g^	2.60 ± 0.01^a^	1.36 ± 0.03^a^	8.59 ± 0.05^e^
dichlorogenic acids	97.42 ± 2.39^h^	10.27 ± 1.35^a^	–16.58 ± 0.10^b^	–29.77 ± 0.08^d^	42.67 ± 1.15^g^	96.34 ± 0.12^g^	0.63 ± 0.06^b^	1.09 ± 0.01^b^
caffeine	22.09 ± 1.82^c^	42.27 ± 1.18^d^	–0.33 ± 0.01^c^	–15.28 ± 0.01^a^	48.36 ± 1.05^g^	24.96 ± 0.08^h^	0.50 ± 0.01^f^	3.28 ± 0.01^c^
Dark-Roasted Robusta								
monochlorogenic acids	44.11 ± 6.98^g^	22.67 ± 0.81^e^	–0.35 ± 0.02^c^	–16.87 ± 0.01^a^	53.44 ± 1.20^a^	9.89 ± 0.04^c^	0.35 ± 0.03^c^	10.16 ± 0.02^e^
dichlorogenic acids	12.22 ± 4.45^f^	81.83 ± 1.10^g^	–12.81 ± 0.10^d^	–29.35 ± 0.06^d^	53.50 ± 1.23^a^	77.64 ± 0.01^g^	0.31 ± 0.02^c^	1.46 ± 0.01^b^
caffeine	10.05 ± 2.55^f^	95.23 ± 1.33^g^	–6.70 ± 0.01^e^	–27.34 ± 0.03^c^	66.76 ± 1.10^e^	25.94 ± 0.02^h^	0.71 ± 0.01^b^	2.01 ± 0.01^c^

a Values are expressed as mean ± SD; *n* = 6; the different letters “a–h” in one column correspond to significant differences (*P* < 0.05).

Monochlorogenic acids were characterized by an average lower *K*_a_ value compared with caffeine and dichlorogenic acids. The highest binding constants were found for the fractions of dark-roasted Robusta. All fractions isolated from coffee extracts showed exothermic interactions with AChE, negative Δ*H* values, and positive entropy changes.

The reaction enthalpy determined in the case of the dichlorogenic acid fractions confirmed the most effective formation of the complexes with the enzyme by these compounds, in particular those isolated from green Robusta (−17.58 kJ/mol), and as roasting progressed, a decrease in the interaction enthalpy was observed. High values of the affinity of dichlorogenic acid fractions from Robusta to AChE determined in ITC tests were also observed (ranging from −29.01 to −29.77 kJ/mol), compared to other fractions, which was confirmed by the results obtained using the docking simulation method (Table 1).

Among Arabica fractions, monochlorogenic acids from the light- and dark-roasted beans had a relatively high affinity for AChE, from −29.35 to 32.53 kJ/mol. Monochlorogenic acids were characterized by an average lower *K*_a_ value compared to caffeine and dichlorogenic acids. The lowest binding constants were found in the fraction of light-roasted Arabica with the highest found in the fraction of dark-roasted Robusta. All fractions isolated from coffee extracts showed exothermic interactions with AChE, a negative Δ*H* value, and a positive entropy change.

The fractions of coffee extracts bound to AChE were competitive inhibitors. They were characterized by varying *K*_i_ values in the range of 0.11–1.67 μmol/L for the monochlorogenic acid fraction of dark-roasted Arabica and the caffeine fraction of green Robusta. Extracts from light- and dark-roasted Robusta and Arabica were characterized by lower *K*_i_ values, below 1 μmol/L. The studies showed that the IC50 values of the monochlorogenic acid fractions isolated from coffee extracts were statistically higher compared to other fractions. It was observed that the concentration of the dichlorogenic acid fraction below 1.5 μmol/μmol enzyme was sufficient to reduce the AChE activity by half, while the concentration of the caffeine fractions was from 2 to 40 μmol/μmol enzyme, while the concentration of monochlorogenic acids was as high as from 4 to 99 μmol/μmol enzyme. Comparing both types of coffee, on average, the fractions obtained from Robusta extracts were characterized by a lower dose necessary to reduce AChE activity by 50%.

Molecular docking was performed to compare the affinity of coffee fractions for AChE. In one simulation, three compounds from one fraction were docked, in different orders. Monochlorogenic acids in coffee occur mainly as 3CQA, 4CQA, and 5CQA and dichlorogenic acids occur as 3,4-DCQA, 3,5-DCQA, and 4,5-DCQA. Different binding modes of given compounds were tested during docking simulations. In Table 4, three steps are presented that represent the docking of the first polyphenol to the enzyme in the presence of the others, and additionally, the average binding energy is taken. The method uses three docking steps because, with a large number of rotatable bonds, combining compounds at one time could result in a negative docking algorithm. Single docking cycles were used to increase the accuracy of the results. Each new rotatable bond added increases the degree of freedom of the ligand, which then leads to an increase in possible conformations. Depending on the algorithm used, there may be differences in results. The extended conformation space usually shows less accurate results.^52^ Therefore, we can observe in Table 4 that LF docking shows higher affinity values than AD. The binding energy for the LF method ranged from −37.72 to −44.94 kJ/mol, for the conformations 3-CQA, 5-CQA, and 4-CQA and 4,5-DCQA, 3,5-DCQA, and 3,4-DCQA (Table 4). In the case of the AD method, the range was from 29.28 kJ/mol for 3-CQA, 4-CQA, and 5-CQA to −35.34 kJ/mol for 3,4-DCQA, 3,5-DCQA, and 4,5-DCQA. Docking simulation of monochlorogenic acids showed that introducing three compounds into the system with AChE increased the affinity for the enzyme (Table 4) than in the case of single compounds (Table 1), even though the number of active sites was the same as when only one compound was docked. However, in the case of dichlorogenic acids, the affinity of the mixture was lower, except for the combination of 4,5-DCQA, 3,4-DCQA, and 3,5-DCQA, which showed Δ*G* amounting to 47.52 kJ/mol. To check the docking protocol done, we carried out the same protocol for a known inhibitor for AChE as E20; also, the crystallographic pose is provided in the same PDB structure (1EVE). The results obtained in both algorithms demonstrate that this protocol works, obtaining an even higher binding score than the rest of the molecules tested by this workflow (−46.02 kJ/mol for AD and −40.04 kJ/mol for LF).

**Table 4:** Binding Energy (the Affinity for AChE) Calculated from the Docking Simulation by LeadFinder and AutoDock Vina Software

	energy (kJ/mol)			
sequence	step 1 (LF/AD)	step 2 (LF/AD)	step 3 (LF/AD)	medium (LF/AD)
3CQA,4CQA,5CQA	–47.31/–37.05	–38.69/–24.20	–41.37/–26.59	–42.46/–29.28
3CQA,5CQA,4CQA	–47.31/–37.05	–29.64/–23.86	–36.22–28.72	–37.72/–29.88
4CQA,3CQA,5CQA	–46.31/–37.56	–33.45/–27.26	–44.05/–27.13	–41.27/–30.65
4CQA,5CQA,3CQA	–46.31/–37.56	–30.81/–25.87	–42.91/–28.14	–40.01/–30.52
5CQA,3CQA,4CQA	–45.22/–39.48	–40.65/–27.84	–37.26/–21.39	–41.04/–29.57
5CQA,4CQA,3CQA	–45.22/–39.48	–40.15/–27.84	–28.30/–24.07	–37.89/–30.47
3,4-DCQA,3,5-DCQA,4,5-DCQA	–53.36/–45.13	–48.44/–31.82	–32.41/–29.06	–44.83/–35.34
3,4-DCQA,4,5-DCQA,3,5-DCQA	–53.36/–45.13	–41.91/–26.00	–36.13/–27.09	–43.89/–32.74
3,5-DCQA,3,4-DCQA,4,5-DCQA	–48.99/–45.38	–38.43/–25.20	–35.04/–25.62	–40.82/–32.07
3,5-DCQA,4,5-DCQA,3,4-DCQA	–48.99/–45.38	–42.83/–27.13	–31.15/–24.12	–40.99–32.21
4,5-DCQA,3,4-DCQA,3,5-DCQA	–54.39/–43.38	–38.06/–25.96	–37.14/–19.64	–43.20/–29.66
4,5-DCQA,3,5-DCQA,3,4-DCQA	–54.39/–43.38	–47.52/–32.78	–32.91/–23.40	–44.94/–33.19

Docking several compounds, in this case several compounds most significant for a given CHA fraction, shows that different isomers, binding in different ways to the enzyme (Figures 6 and 7), can stabilize the complex formed in the active site of AChE. This supports the view that mixtures of compounds may act synergistically in various signaling pathways and metabolism and be more effective as therapeutics than single compounds.

**Figure 2:**
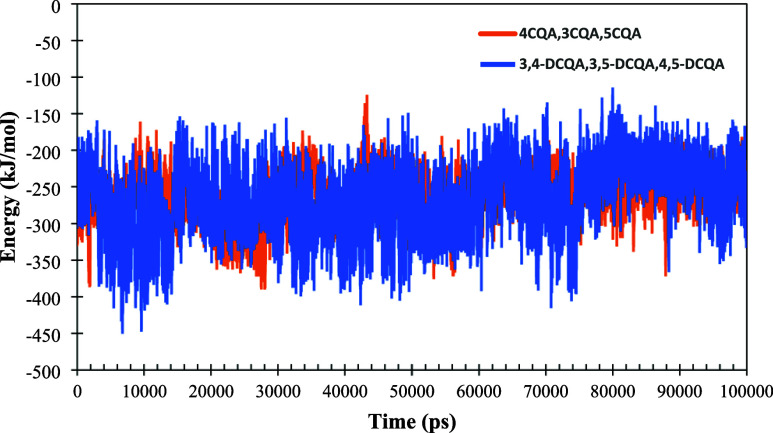
MM-PBSA analysis results for 4CQA, 3CQA, and 5CQA (orange) and 3,4-DCQA, 3–5-DCQA, and 4,5-DCQA (blue) binding modes. The graph shows the total energy (kJ/mol) obtained for each frame in the 100 ns simulation performed for each binding modes.

After obtaining the docking results, we performed MD simulations to calculate MM-PBSA [TC3](Figure 2) and root mean square deviation (RMSD) (Figures 8 and 9) for the complexes with the binding modes of higher affinity results between both methods: 4CQA, 3CQA, and 5CQA and 3,4-DCQA, 3,5-DCQA, and 4,5-DCQA. The clusters more stable by RMSD for the best binding mode for monochlorogenic and dichlorogenic acids obtain −250.41 and −263.03 kJ/mol, respectively. These values are much higher than the binding energy values obtained by docking, indicating the importance of the solvation factor, and corroborate the high affinity of this complex during the simulation time. Finally, the energies were decomposed by residues, highlighting the importance of MET83 (−17.28 kJ/mol) and GLU445 (−11.64 kJ/mol) for monochlorogenic acids and TYR334 (−22.65 kJ/mol), VAL71 (−26.92 kJ/mol), and GLN74 (−11.87 kJ/mol) for dichlorogenic acids, which exhibited the highest energy values within the active site. These residues were identified as key to the interaction in both techniques, docking and molecular dynamics.

It can be concluded that the isolated fractions from coffee extracts, which were interacted with AChE, increased their affinity for the enzyme in the case of monochlorogenic acids along with the degree of roasting for Arabica, while for Robusta, a decrease in the affinity with the degree of roasting was observed (Table 3). However, differences in the affinity reflecting the degree of roasting for the dichlorogenic acid fractions for Robusta were statistically insignificant and remained at the level of 29 kJ/mol, while for Arabica, the affinity for AChE decreased for light-roasted coffee and further increased for dark-roasted coffee (Table 3).

**Figure 3:**
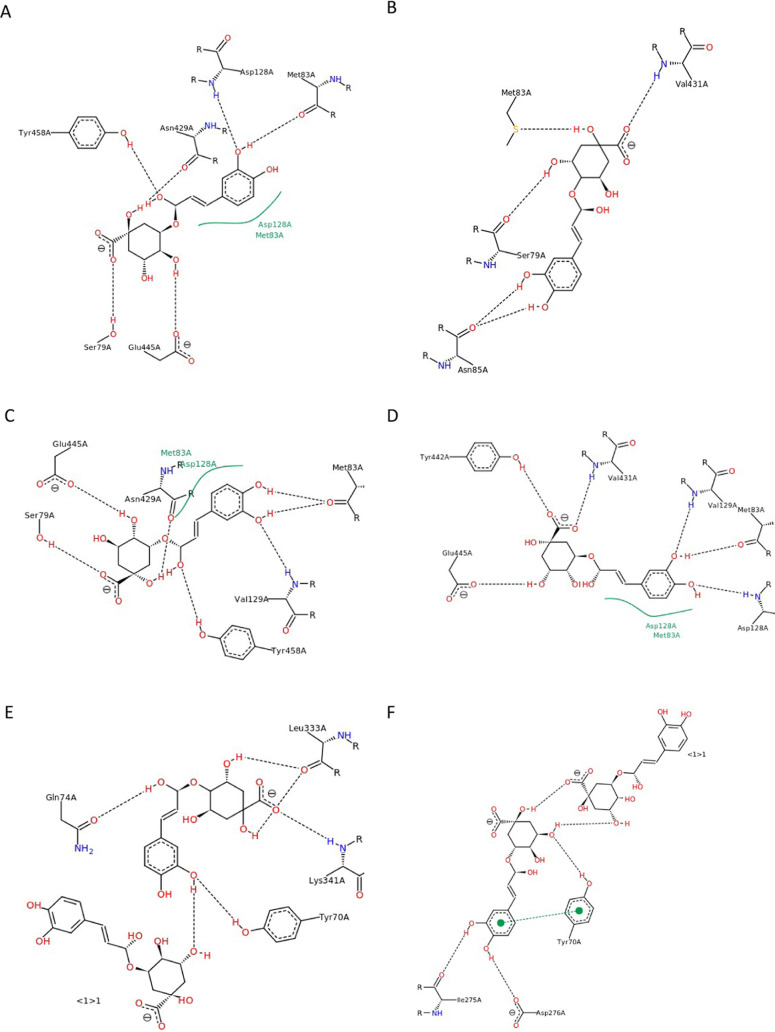

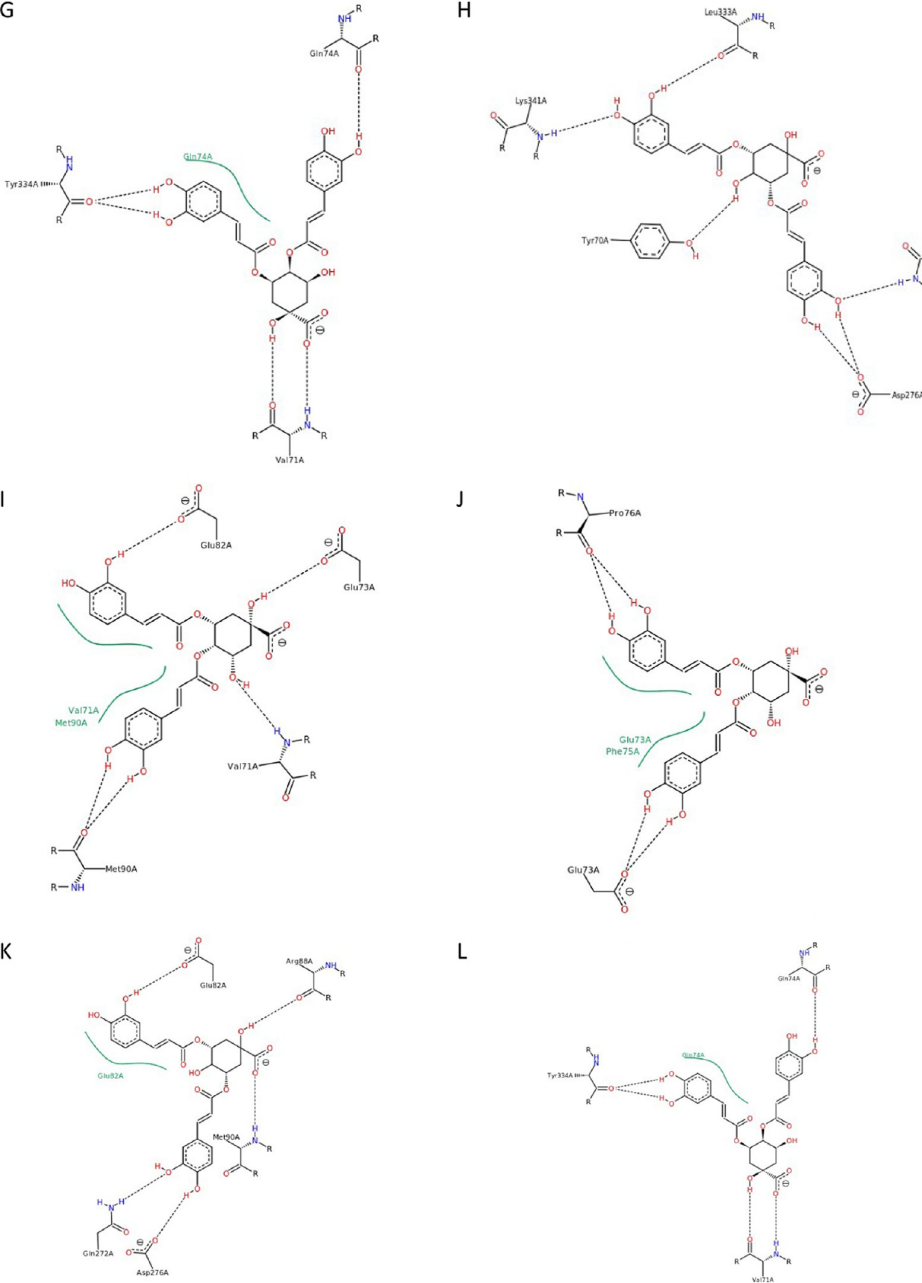
Docking simulation results, 2D models, and interactions with AChE of mixtures of CHAs docked in different orders. (A) 3-CQA, 4-CQA, 5-CQA; (B) 3-CQA, 5-CQA, 4-CQA; (C) 4-CQA, 3-CQA, 5-CQA; (D) 4-CQA, 5-CQA, 3-CQA; (E) 5-CQA, 3-CQA, 4-CQA; (F) 5-CQA, 4-CQA, 3-CQA; (G) 3,4-DCQA, 3,5-DCQA, 4,5-DCQA; (H) 3,4-DCQA, 4,5-DCQA, 3,5-DCQA; (I) 3,5-DCQA, 3,4-DCQA, 4,5-DCQA; (J) 3,5-DCQA, 4,5-DCQA, 3,4-DCQA; (K) 4,5-DCQA, 3,4-DCQA, 3,5-DCQA; and (L) 4,5-DCQA, 3,5-DCQA, 3,4-DCQA. Black dashed lines show hydrogen bonds, green dashed lines show π–π interactions, and continuous green lines represent hydrophobic interactions.

The order of docking monochlorogenic acids 5-CQA, 4-CQA, and 3-CQA resulted in the highest affinity for the active site of AChE. This is related to the occurrence of π–π interactions with TYR72 and five hydrogen bonds, including three direct bonds of AChE with polyphenol and two between polyphenols [TC5](Figure 3F and Table 4). However, for dichlorogenic acids, the highest affinity for AChE was calculated for the docking in the order 4,5-DCQA, 3,4-DCQA, and 3,5-DCQA, which is characterized by hydrophobic interactions involving GLU82 and five hydrogen bonds (Figure 3K).

In the case of fractions isolated from coffee extracts and after large intestine digestion *in vitro*, the binding constant was characterized by 20% higher values compared to the constant before the digestion (Table 3). The interactions of the digested fractions isolated from coffee extracts with the enzyme, regardless of the digestion stage, were exothermic. As a result of digestion, caffeic acid was reduced by 10% in the case of digestion without bacteria and by 5% in the presence of probiotic bacteria.^23^ After digestion in the stomach and small intestine, the enthalpies of interactions of the digested fractions with AChE, as well as the Gibbs free energy, were significantly reduced compared to the values before digestion. The enthalpy change Δ*H* of interactions after gastric digestion ranged from −40.96 to −10.12 kJ/mol for the caffeine fraction isolated from green Robusta and monochlorogenic acids isolated from green Arabica. After digestion in the small intestine, the enthalpy of interactions was almost equal among fractions, in the range of −32–31 kJ/mol. After digestion in the large intestine, Δ*H* increased by approximately 60–75%, while further digestion in the colon decreased Δ*H* to the range of −2.18 to −30.11 kJ/mol.

**Figure 4:**
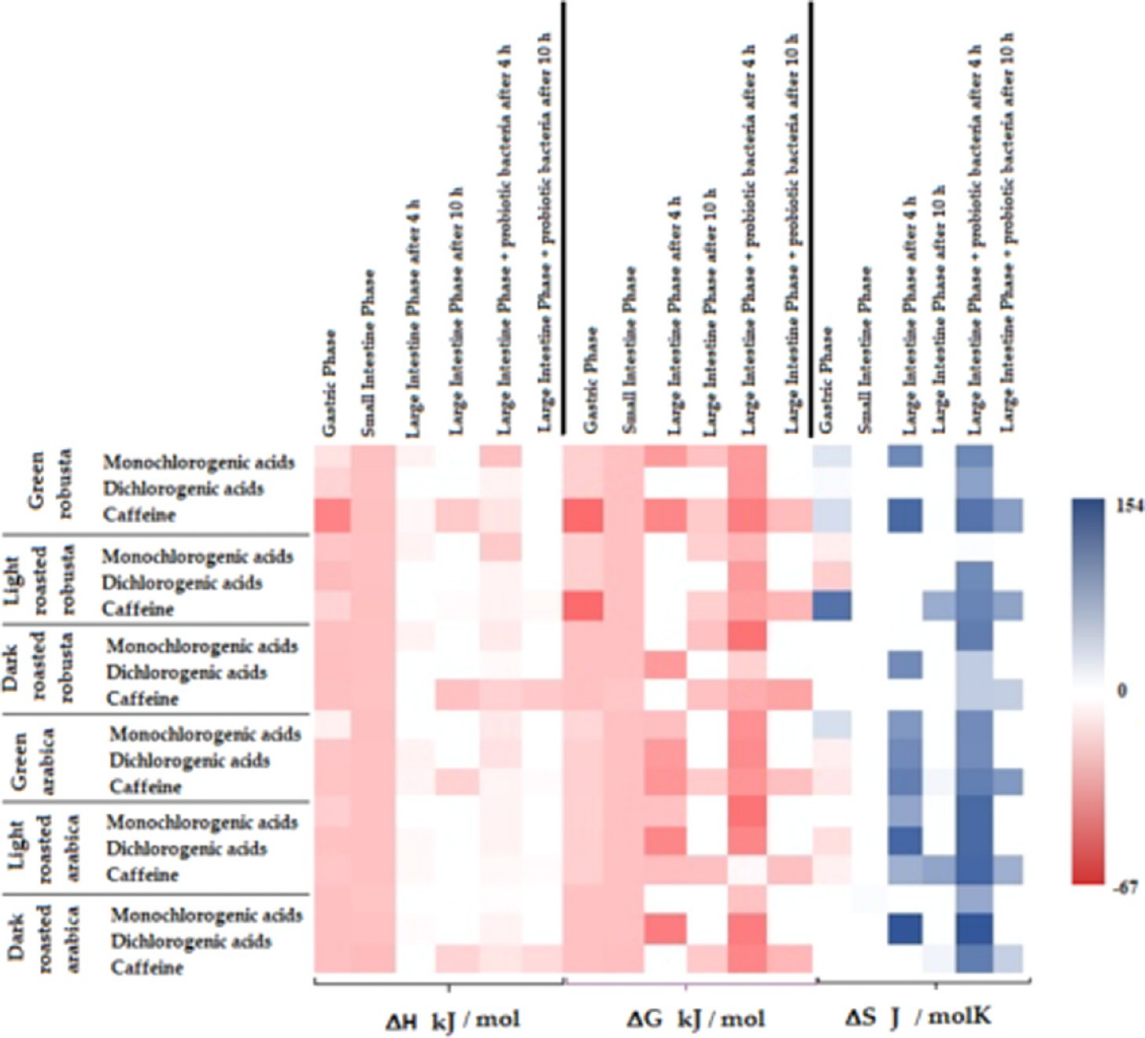
Thermodynamic parameters of the interactions of fractions isolated from coffee extracts digested *in vitro* with AChE.

The presence of probiotic bacterial strains during digestion in the large intestine resulted in a higher reaction enthalpy and affinity for AChE of all fractions obtained from the coffee extracts. After digestion in the large intestine, monochlorogenic acids had an affinity for the enzyme of −67 kJ/mol. The results of the analysis are presented in Figure 4.

The *K*_i_ measurements of *in vitro* digested fractions isolated from coffee extracts that interacted with AChE indicated that they bound to the enzyme as competitive inhibitors and were characterized by similar and low *K*_i_ values in the range of 0.22–1.25 μmol/L for the dichlorogenic acid fraction isolated from green Robusta extract after digestion in the large intestine in the presence of probiotic bacteria and monochlorogenic acid fraction from dark-roasted Arabica after digestion in the small intestine (Table 5).

**Table 5:** Inhibition Constant of Fractions Isolated from Coffee Extracts of Hydrolysis of ACh with AChE^a^

		*K*_i_ (μmol/L)		
		*K*_M_ ACh 48.5 μmol/L		
fractions of coffee extracts		monochlorogenic acid	dichlorogenic acid	caffeine
gastric phase	green robusta	0.31 ± 0.01^a^	0.30 ± 0.01^a^	0.32 ± 0.01^a^
	light-roasted robusta	0.36 ± 0.01^a^	0.34 ± 0.01^a^	0.33 ± 0.01^a^
	dark-roasted robusta	0.51 ± 0.02^b^	0.52 ± 0.01^b^	0.54 ± 0.02^b^
	green arabica	0.44 ± 0.02^c^	0.45 ± 0.02^c^	0.34 ± 0.01^a^
	light-roasted arabica	0.61 ± 0.01^d^	0.59 ± 0.01^b^	0.36 ± 0.01^a^
	dark-roasted arabica	1.12 ± 0.01^e^	1.05 ± 0.03^d^	0.64 ± 0.02^c^
small intestine phase	green robusta	0.34 ± 0.01^a^	0.32 ± 0.01^a^	0.33 ± 0.01^a^
	light-roasted robusta	0.38 ± 0.01^a^	0.34 ± 0.01^a^	0.34 ± 0.01^a^
	dark-roasted robusta	0.52 ± 0.02^b^	0.53 ± 0.02^b^	0.55 ± 0.03^b^
	green arabica	0.45 ± 0.03^c^	0.47 ± 0.02^c^	0.35 ± 0.01^a^
	light-roasted arabica	0.63 ± 0.03^d^	0.61 ± 0.03^e^	0.37 ± 0.01^a^
	dark-roasted arabica	1.25 ± 0.02^e^	1.09 ± 0.03^d^	0.65 ± 0.03^c^
large intestine phase 4 h	green robusta	0.36 ± 0.01^a^	0.37 ± 0.01^a^	0.35 ± 0.01^a^
	light-roasted robusta	0.39 ± 0.01^a^	0.35 ± 0.01^a^	0.36 ± 0.01^a^
	dark-roasted robusta	0.53 ± 0.02^b^	0.58 ± 0.02^b^	0.57 ± 0.03^b^
	green arabica	0.46 ± 0.02^c^	0.49 ± 0.02^c^	0.35 ± 0.01^a^
	light-roasted arabica	0.65 ± 0.03^d^	0.68 ± 0.02^e^	0.37 ± 0.01^a^
	dark-roasted arabica	1.24 ± 0.05^e^	1.11 ± 0.01^d^	0.66 ± 0.03^c^
large intestine phase 4 h + probiotic bacteria	green robusta	0.32 ± 0.01^a^	0.22 ± 0.00^f^	0.34 ± 0.01^a^
	light-roasted robusta	0.34 ± 0.01^a^	0.31 ± 0.01^a^	0.35 ± 0.01^a^
	dark-roasted robusta	0.49 ± 0.01^c^	0.52 ± 0.02^b^	0.55 ± 0.02^b^
	green arabica	0.44 ± 0.02^c^	0.40 ± 0.01^c^	0.33 ± 0.01^a^
	light-roasted arabica	0.61 ± 0.02^d^	0.63 ± 0.03^e^	0.34 ± 0.01^a^
	dark-roasted arabica	1.21 ± 0.03^e^	1.04 ± 0.03^d^	0.62 ± 0.03^c^
large intestine phase 10 h	green robusta	0.34 ± 0.01^a^	0.39 ± 0.01^a^	0.35 ± 0.02^a^
	light-roasted robusta	0.38 ± 0.01^a^	0.37 ± 0.01^a^	0.37 ± 0.02^a^
	dark-roasted robusta	0.52 ± 0.02^b^	0.61 ± 0.02^e^	0.59 ± 0.01^b^
	green arabica	0.45 ± 0.01^c^	0.52 ± 0.02^b^	0.36 ± 0.02^a^
	light-roasted arabica	0.64 ± 0.03^d^	0.69 ± 0.03^e^	0.38 ± 0.02^a^
	dark-roasted arabica	1.23 ± 0.03^e^	1.16 ± 0.03^d^	0.67 ± 0.03^c^
large intestine phase 10 h + probiotic bacteria	green robusta	0.29 ± 0.01^a^	0.35 ± 0.01^a^	0.30 ± 0.01^a^
	light-roasted robusta	0.30 ± 0.01^a^	0.32 ± 0.01^a^	0.31 ± 0.02^a^
	dark-roasted robusta	0.43 ± 0.01^c^	0.54 ± 0.01^b^	0.54 ± 0.02^b^
	green arabica	0.41 ± 0.02^c^	0.49 ± 0.02^c^	0.32 ± 0.02^a^
	light-roasted arabica	0.58 ± 0.01^b^	0.62 ± 0.04^e^	0.32 ± 0.01^a^
	dark-roasted arabica	1.16 ± 0.03^e^	1.03 ± 0.05^d^	0.60 ± 0.02^c^

a Values are expressed as mean ± SD; *n* = 6; different letters “a–f” in one column correspond to significant differences (*P* < 0.05).

**Figure 5:**
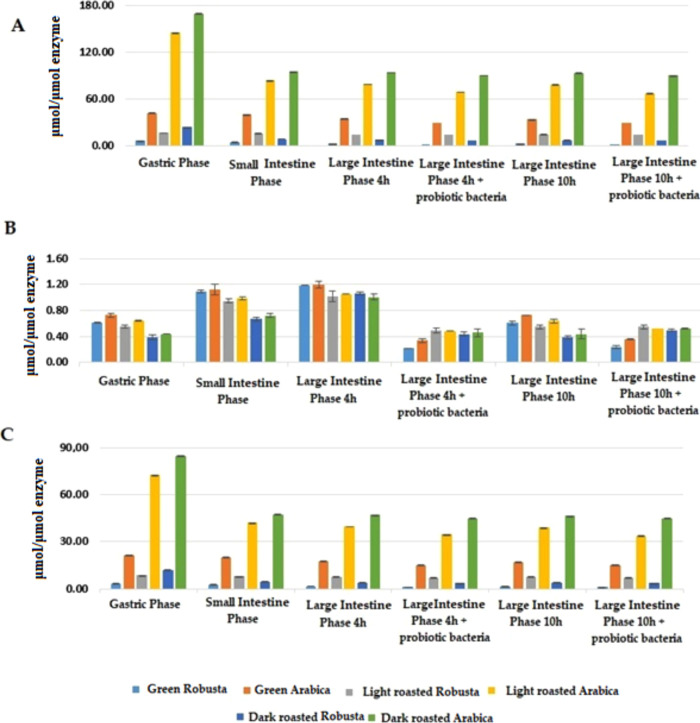
Concentration of bioactive compounds of (A) monochlorogenic acid; (B) dichlorogenic acid; and (C) caffeine fractions isolated from coffee extracts digested *in vitro* causing a decrease in AChE activity by 50%.

**Figure 6:**
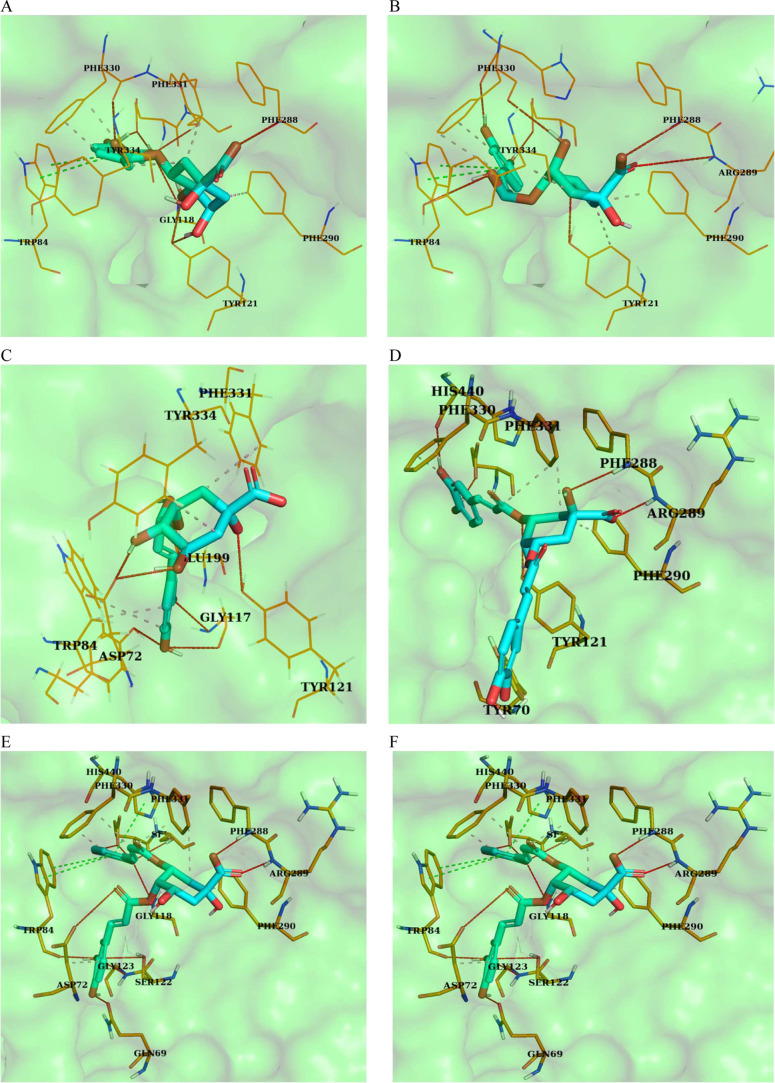
Three-dimensional (3D) model of the docking calculation results by software with the best score for each compound. These show the interactions of AChE with monochlorogenic and dichlorogenic acids; red line: hydrogen bonds and pink dashed line: hydrophobic interactions. (A) 3-CQA, (B) 4-CQA, (C) 5-CQA, (D) 3,5-DCQA, (E) 4,5-DCQA, and (F) 3,4-DCQA.

**Figure 7:**
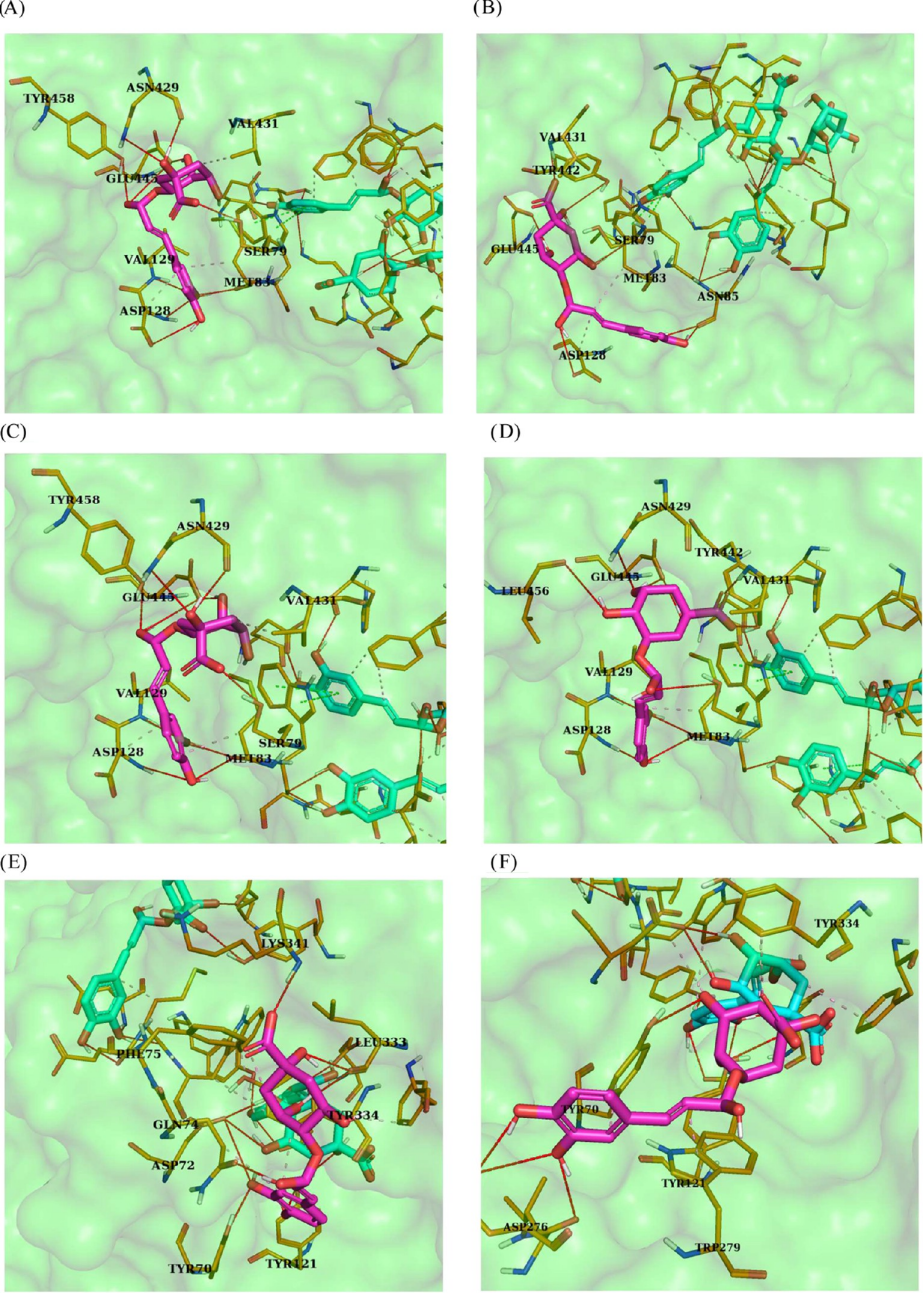

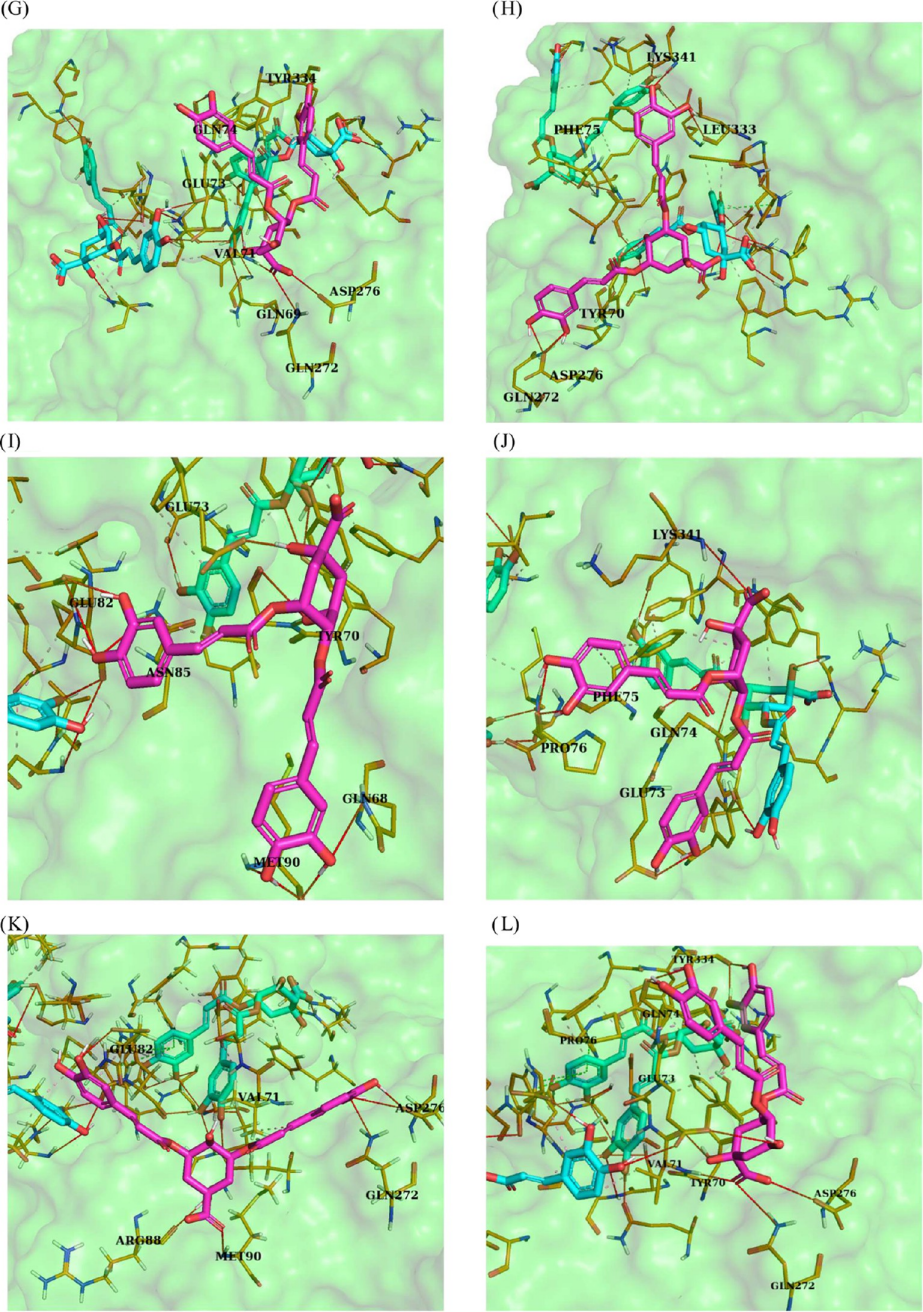
Docking simulation results, 3D models, interactions with AChE of mixtures of CHAs docked in different orders. (A): 3-CQA, 4-CQA, 5-CQA; (B): 3-CQA, 5-CQA, 4-CQA; (C): 4-CQA, 3-CQA, 5-CQA; (D) 4-CQA, 5-CQA, 3-CQA; (E) 5-CQA, 3-CQA, 4-CQA; (F) 5-CQA, 4-CQA, 3-CQA; (G) 3,4-DCQA, 3,5-DCQA, 4,5-DCQA; (H) 3,4-DCQA, 4,5-DCQA, 3,5-DCQA; (I) 3,5-DCQA, 3,4-DCQA, 4,5-DCQA; (J) 3,5-DCQA, 4,5-DCQA, 3,4-DCQA; (K) 4,5-DCQA, 3,4-DCQA, 3,5-DCQA; and (L) 4,5-DCQA, 3,5-DCQA, 3,4-DCQA. Red line: hydrogen bonds, and pink dashed line: hydrophobic interactions.

**Figure 8:**
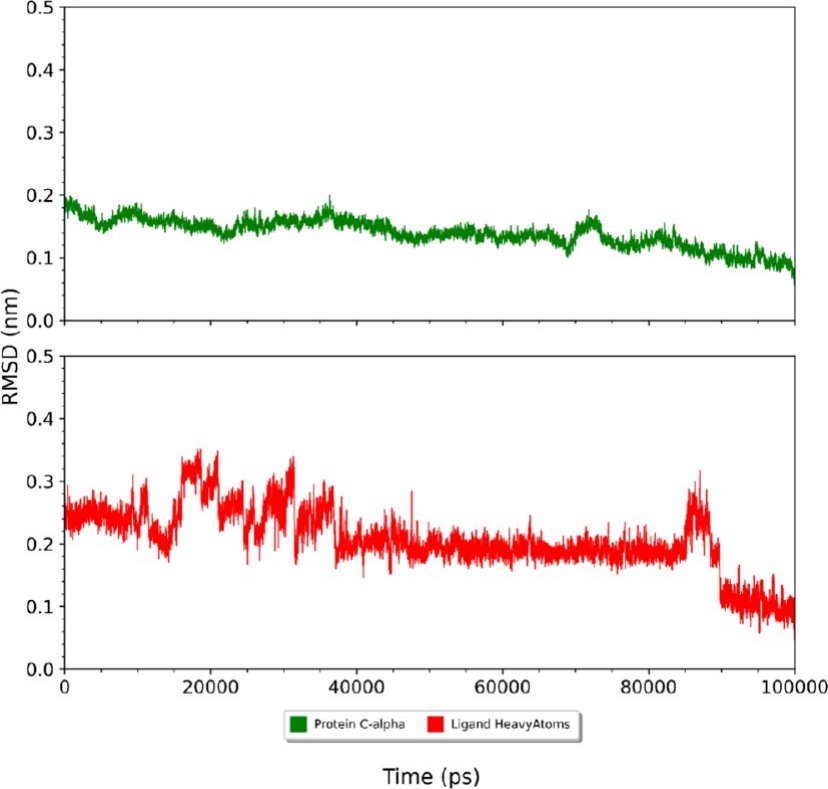
RMSD during MD simulation for the protein C-α backbone (green) and ligand heavy atoms (red) for the 4CQA, 3CQA, and 5CQA and AChE complex.

**Figure 9:**
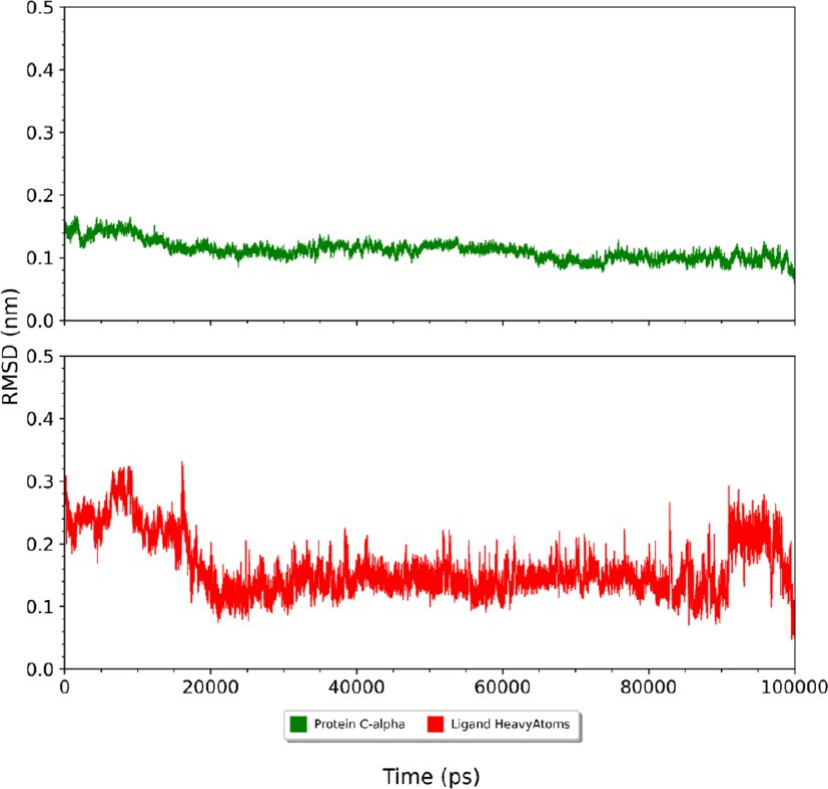
RMSD during MD simulation for the protein C-α backbone (green) and ligand heavy atoms (red) for the 3,4-DCQA, 3–5-DCQA, and 4,5-DCQA and AChE complex.

The studies showed that the IC50 value of digested fractions from coffee extracts was statistically lower in the case of the dichlorogenic acid fraction compared to other fractions isolated from coffee extracts (Figure 5). In turn, the monochlorogenic acid fractions showed twice as high concentrations of compounds inhibiting AChE activity after each digestion stage compared to the caffeine fractions. The concentration of bioactive compounds of the monochlorogenic acid fractions causing a reduction in the activity of the AChE by half showed a decreasing tendency with the progress of digestion. The highest concentration was demonstrated after digestion in the gastric phase, with the lowest found after digestion in the large intestine with the participation of selected strains of probiotic bacteria. Comparing the degree of roasting and coffee species, it can be seen that the most effective AChE inhibitors were Robusta coffee fractions, especially from green beans. Fractionated extracts from dark- and light-roasted Arabica showed high IC50 values and were less effective AChE inhibitors. Dichlorogenic acid fractions had very significantly lower IC50 values and the differences were especially seen in the case of Arabica. This is related to the structure of caffeic acid diesters and their higher affinity for the enzyme, resulting among others from the higher values of energy contribution of salt bridges, which was confirmed by molecular modeling (Figures 6–9).

It was observed that the fractions of dichlorogenic acids isolated from coffee extracts bound to AChE most effectively, which is also confirmed by previous studies of the ITC parameters of the fraction before digestion and studies of standard substances and their molecular modeling with the enzyme. After digestion in the stomach, the concentration of effective inhibitors expressed as IC50 was in the range of 0.39–0.73 μmol/μmol enzyme, for dichlorogenic acids isolated from the extract of dark-roasted Robusta and green Arabica. Among the analyzed fractions of dichlorogenic acids isolated from coffee extracts, the lowest IC50 value was recorded for green Robusta after digestion in the large intestine in the presence of probiotic bacteria with the highest recorded for green coffees of both species after digestion in the small intestine and large intestine without the bacteria. After digestion in the large intestine in the presence of probiotic bacteria, the concentration necessary to reduce AChE activity by 50% decreased by approximately half and was in the range of 0.22–0.49 μmol/μmol enzyme, in the case of the dichlorogenic acid fraction from the green and light-roasted Robusta.

Studies have also shown that caffeine fractions isolated from coffee extracts, unlike CHA fractions, showed a similar level of AChE inhibition after each digestion stage. In general, low concentrations of fractions of Robusta extracts calculated as IC50 were observed. The caffeine fractions showed the highest activity after digestion in the colon in the presence of probiotic bacteria, ranging from 1.23 to 45.01 μmol/μmol enzyme in the case of caffeine and accompanied substances isolated from green Robusta and dark-roasted Arabica. After digestion in the stomach, the IC50 value was 3.44–84.56 μmol/μmol enzyme of the fraction isolated from green Robusta and dark-roasted Arabica. Then, during digestion in the small intestine, the IC50 concentration statistically significantly decreased to the range of 2.60–47.58 μmol/μmol enzyme, for the fraction isolated from green Robusta and dark-roasted Arabica, and then decreased slightly after digestion in the large intestine and in the colon. The IC50 value decreased, although statistically insignificantly, after digestion in the presence of probiotic bacteria, which suggested that the activity of bacteria to change the substances in the caffeine fraction was limited.

Summarizing the conducted research on the interactions and affinity of bioactive components of coffee extracts and their fractions for acetylcholinesterase, it can be concluded that coffee is a good source of AChE inhibitors, and the concentration of bioactive compounds contained in typically consumed amounts of coffee extracts ensures their physiological importance and high effectiveness in inhibiting the activity of the enzyme. In particular, such an effect has the fraction of dichlorogenic acids. The studies indicated that coffee extracts and their fractions after simulated *in vitro* digestion significantly inhibited the hydrolysis of acetylcholine by the enzyme as a result of the formation of complexes in the active sites of AChE.

From the above, it follows that coffee ingredients can be considered compounds that have a beneficial effect on the protection of the neurotransmitter and ensure its required level, which improves cognitive functions in people suffering from dementia. The extract from dark-roasted Arabica after *in vitro* digestion at the stomach stage showed the highest affinity for AChE and formed the most stable complexes with the enzyme. It can also be concluded that the presence of probiotic bacteria in the simulated digestive tract increased the affinity of the digested coffee extracts for the enzyme, which means that microbiota in the gastrointestinal tract may increase the digestibility of polymeric substances, releasing low-molecular weight compounds, and therefore the ability to form complexes in the active site of the enzyme.
